# Quantum-state-dependent decay rates of electrostatically trapped Rydberg NO molecules

**DOI:** 10.1039/d1cp01930a

**Published:** 2021-07-22

**Authors:** M. H. Rayment, S. D. Hogan

**Affiliations:** Department of Physics and Astronomy, University College London Gower Street London WC1E 6BT UK s.hogan@ucl.ac.uk

## Abstract

Nitric oxide (NO) molecules travelling in pulsed supersonic beams have been prepared in long-lived Rydberg–Stark states by resonance-enhanced two-colour two-photon excitation from the X ^2^Π_1/2_ (*v*′′ = 0, *J*′′ = 3/2) ground state, through the A ^2^Σ^+^ (*v*′ = 0, *N*′ = 0, *J*′ = 1/2) intermediate state. These excited molecules were decelerated from 795 ms^−1^ to rest in the laboratory-fixed frame of reference, in the travelling electric traps of a transmission-line Rydberg–Stark decelerator. The decelerator was operated at 30 K to minimise effects of blackbody radiation on the molecules during deceleration and trapping. The molecules were electrostatically trapped for times of up to 1 ms, and detected *in situ* by pulsed electric field ionisation. Measurements of the rate of decay from the trap were performed for states with principal quantum numbers between *n* = 32 and 50, in Rydberg series converging to the *N*^+^= 0, 1, and 2 rotational states of NO^+^. For the range of Rydberg states studied, the measured decay times of between 200 μs and 400 μs were generally observed to reduce as the value of *n* was increased. For some particular values of *n* deviations from this trend were seen. These observations are interpreted, with the aid of numerical calculations, to arise as a result of contributions to the decay rates, on the order of 1 kHz, from rotational and vibrational channel interactions. These results shed new light on the role of weak intramolecular interactions on the slow decay of long-lived Rydberg states in NO.

## Abstract 2

Nitric oxide molecules in high Rydberg states have been decelerated and electrostatically trapped in a chip-based Rydberg–Stark decelerator. Measured trap decay rates shed new light on slow decay processes of Rydberg states in small molecules.

## Graphical abstract

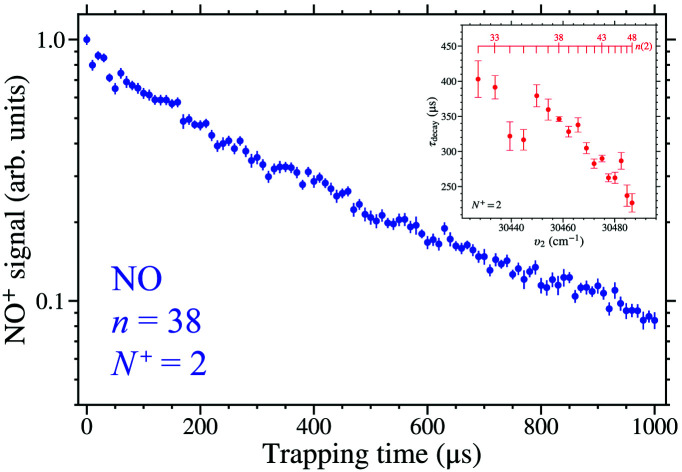

## 1 Introduction

Rydberg states of atoms and small molecules, including H_2_, N_2_, O_2_, and NO, are of importance in recombination processes that occur in laboratory, atmospheric, and astrophysical plasmas.^1–6^ They are also of interest in studies of chemical dynamics at low temperatures. For example, they can be employed to study ion–molecule reactions, as most recently demonstrated in the case of the H_2_^+^ + HD → H_2_D^+^ + H reaction, at temperatures as low as 50 mK.^7–10^ In this setting the Rydberg electron shields the reaction centre from stray electric fields that would otherwise cause heating. Long-lived Rydberg states of small molecules also offer opportunities for studies of low-energy molecular scattering that are dominated at long range by resonant dipole–dipole interactions,^11^ and the exploration of the chemistry associated with ultra-long range Rydberg molecules^12^ formed of a Rydberg atom or molecule bound to a ground-state atom or molecule through low-energy Rydberg-electron scattering.^13,14^ They are also important in tests of molecular quantum mechanics and quantum chemistry, for example, through the precise determination of ionisation and dissociation energies.^15–17^ The implementation of general methods for preparing samples of cold molecules in high Rydberg states is of interest in each of these areas, and is also foreseen as a means of producing cold samples of ground-state species following de-excitation.^18^ For each of these endeavours a detailed understanding of the lifetimes and decay processes of long-lived Rydberg states is essential.

The large electric dipole moments associated with Rydberg–Stark states of atoms and molecules mean that forces can be exerted on them using inhomogeneous electric fields.^19,20^ This has led to the development of the methods of Rydberg–Stark deceleration^21–23^ and the implementation of guides,^24–27^ beam-splitters,^28^ decelerators,^29–31^ and traps^18,31–36^ for atoms and molecules. To implement these experimental techniques it is necessary to prepare Rydberg states with sufficiently long lifetimes, *i.e.*, >10 μs, and small avoided crossing between individual sublevels in the presence of electric fields, that can be traversed diabatically on typical experimental timescales. Both of these requirements can be met in general for small molecules through the preparation of Rydberg states with values of |*M*_*N*_| ≥ 3 (*M*_*N*_ is the projection of the total angular momentum vector excluding spin, *N⃑*, onto the *z*-axis in the laboratory frame of reference).^18^ In previous experiments with H_2_, the preparation of selected |*M*_*N*_| = 3 Rydberg–Stark states was achieved by three-photon excitation from the X ^1^Σ_g_^+^(*v* = 0, *J* = 0) ground electronic state using circular polarised vacuum ultraviolet, visible, and infrared laser radiation.^18,37^ When these molecules were confined in a cryogenically cooled trap, *n*-dependent (*n* is the principal quantum number) decay times on the order of 100 μs, and effects of rotational channel interactions, were observed.^38^ In the work described here, |*M*_*N*_| ≥ 3 Rydberg states were prepared in NO by two-photon laser photoexcitation from the ground electronic state with subsequent *M*_*N*_-mixing arising as a result of intramolecular interactions and effects of static and time-varying electric fields close to the time of photoexcitation.^39^ Deceleration and trapping of the NO molecules in these long-lived states has permitted studies of slow decay processes over timescales of up to 1 ms that were previously inaccessible.

Studies of the lifetimes, and decay dynamics of high-*n*, *n* = 40 to 122, *v*^+^ = 0 (*v*^+^ is the vibrational quantum number of the NO^+^ ion core) Rydberg states in NO have been reported previously by Vrakking and Lee.^40,41^ This work was performed to aid in the interpretation of excited-state lifetimes observed in ZEKE spectroscopy experiments.^42^ These measurements were performed on timescales of up to ∼1 μs. The results showed that *n*p(0) and *n*f(2) [*n*ℓ(*N*^+^), where ℓ is the orbital angular momentum quantum number of the Rydberg electron, and *N*^+^ is the rotational quantum number of NO^+^] Rydberg states in NO have short lifetimes, on the order of ∼1 ns and ∼10 ns, respectively, for this range of values of *n* in zero-electric-field. This indicated that fast predissociation dominated the decay of these low-ℓ states. However, when photoexcitation was performed in the presence of sufficiently large electric fields, lifetimes on the order of 100 ns were observed. Calculations reported by Bixon and Jortner^43^ showed that this lifetime enhancement could be accounted for by considering a combination of electric-field-induced ℓ-mixing and intramolecular interactions, between each optically accessible short-lived low-ℓ state and long-lived high-ℓ states.

Measurements and calculations reported by Goodgame *et al.*^44^ on the Stark effect in *v*^+^ = 1 Rydberg states in NO with values of *n* between 10 and 20, located above the *v*^+^ = 0 series limit, allowed for the study of intramolecular interactions, and of the lifetimes of the states excited to be estimated from the measured spectral linewidths. For Rydberg states in series converging to excited vibrational states of NO^+^, non-radiative decay by vibrational autoionisation can occur if the interaction between the Rydberg electron and the ion core couples the Rydberg state with the continuum of a lower vibrational series. Rydberg states in the *v*^+^ = 1 series, that lie above the *v*^+^ = 0 series limit, can therefore undergo vibrational autoionisation because of coupling to the *v*^+^ = 0 continuum. From a comparison of calculated and measured spectral linewidths, lifetimes of the low-ℓ states with *v*^+^ = 1 and values of *n* between 10 and 20 were estimated to be between ∼1 ps and ∼50 ps. These lifetimes are dominated by fast non-radiative decay and contain contributions from both predissociation and autoionisation.

Further studies of Rydberg states in NO in electric fields were carried out by Jones *et al.*^45^ and Patel *et al.*^46^ to investigate effects of intramolecular interactions on the Stark structure of *v*^+^ = 0 Rydberg states. These works included laser photoexcitation spectra recorded in electric fields of between 0 and 150 V cm^−1^, with detection by pulsed electric field ionisation, and by monitoring predissociated fragments by photoionisation. These measurements, combined with the results of numerical calculations, allowed intramolecular couplings of optically accessible *n*p(*N*^+^) and *n*f(*N*^+^) states with neighbouring states to be studied. They also allowed the distribution of *n*p(*N*^+^) and *n*f(*N*^+^) character among the ℓ-mixed Stark states in the presence of electric fields to be determined. The implementation of two complementary detection methods in these works yielded information on the importance of predissociation in the decay of the states excited, and how this changed in an electric field.

Recently, using a two-dimensional spectroscopy technique based on laser photoexcitation and delayed state-selective electric field ionisation, long-lived Rydberg states in NO were characterised in detail for values of *n* in the range from 40 to 100.^39^ States with lifetimes in excess of 10 μs were identified and observed to ionise diabatically. These results indicated that high-|*M*_*N*_| ℓ-mixed states with long lifetimes, *i.e.*, states without any short-lived ℓ ≤ 3 character, were populated upon photoexcitation. That work led to the first experiments to decelerate and electrostatically trap Rydberg NO molecules.^47^ The experiments and calculations reported here build upon these works, and include new high-precision decay-rate measurements for long-lived Rydberg states of different rotational series, and the identification of contributions from weak intramolecular interactions to the measured decay rates.

In the following, the experimental apparatus and techniques are described in Section 2. In Section 3, calculations of the energy-level structure and lifetimes of the Rydberg states in NO in electric fields, and considerations necessary for the implementation of Rydberg–Stark deceleration are discussed. In Section 4, the results of trap decay measurements performed for hydrogenic Rydberg–Stark states with values of *n* between 32 and 50 are presented and interpreted in terms of the effects of intramolecular rotational and vibrational channel interactions. Finally conclusions are drawn in Section 5.

## 2 Experiment

**Fig. 1:**
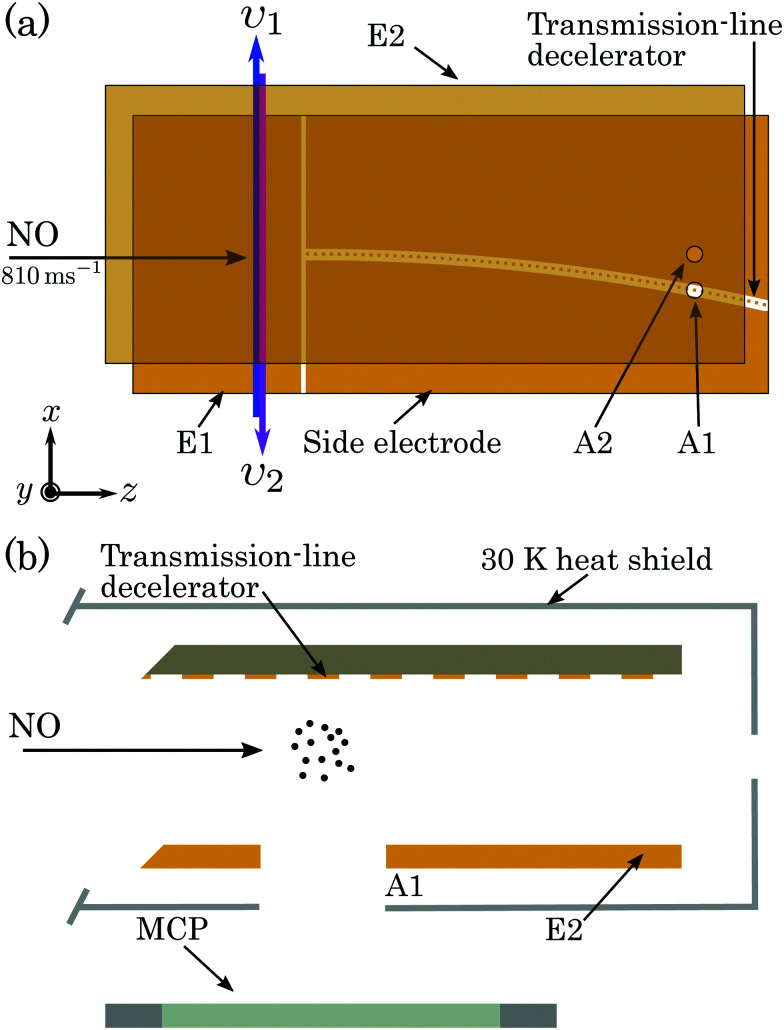
Schematic diagram (not to scale) of the chip-based transmission-line Rydberg–Stark decelerator used in the experiments, (a) in the *xz*-plane, and (b) in the plane perpendicular to the curved decelerator axis close to the detection region. E1 and E2 represent plane metallic electrodes. A1 and A2 are the two detection apertures in electrode E2.

A schematic diagram of the apparatus used in the experiments is shown in Fig. 1. A pulsed supersonic beam of NO was generated with a mean longitudinal speed of 810 ms^−1^. The pulsed valve from which the beam emanated was operated at room temperature at a repetition rate of 25 Hz. After passing through a 2-mm-diameter skimmer, the beam entered the cryogenically cooled region of the apparatus containing a chip-based transmission-line Rydberg–Stark decelerator.^30,33^

**Fig. 2:**
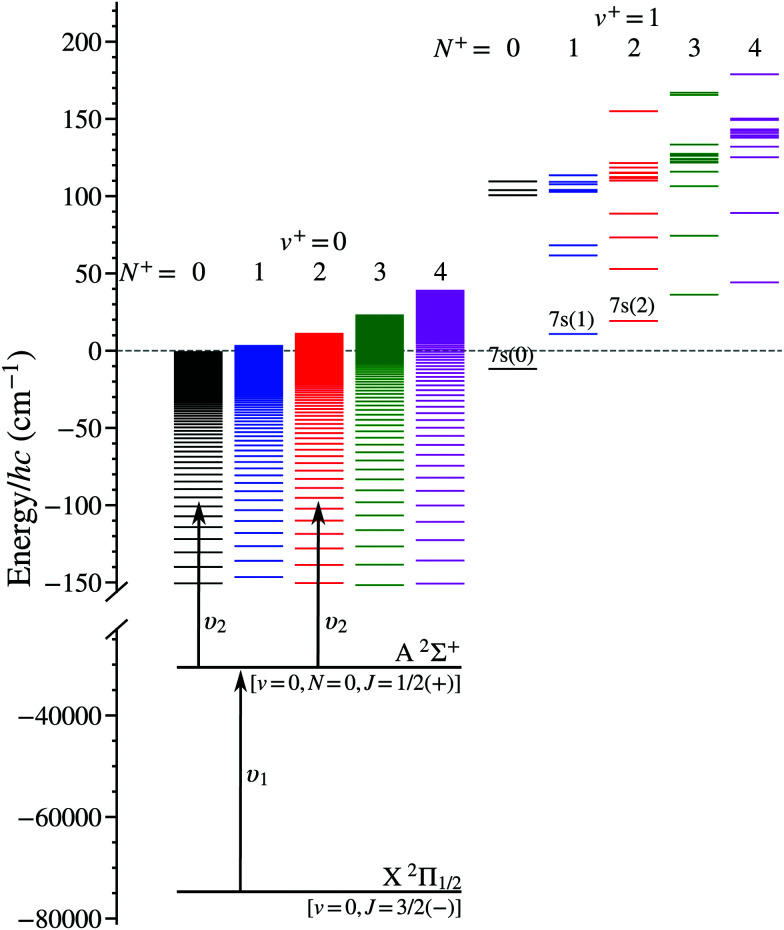
Resonance-enhanced two-colour two-photon excitation scheme employed in the preparation of Rydberg states in NO converging to the lowest vibrational state (*v*^+^ = 0) of the NO^+^ ion core. Low-*n* states in the *v*^+^ = 1 series that are located close to the *v*^+^ = 0, *N*^+^ = 0 series limit are also shown. The energies of the levels in this figure were either measured in the experiments,^39^ or calculated using the methods described in Section 3.1.

Laser photoexcitation of the NO molecules to Rydberg states was performed between electrodes E1 and E2, as shown in Fig. 1(a), using the *n*ℓX^+ 1^Σ^+^(*v*^+^ = 0, *N*^+^) ← A ^2^Σ^+^(*v*′ = 0, *N*′ = 0, *J*′ = 1/2) ← X ^2^Π_1/2_(*v*′′ = 0, *J*′′ = 3/2) resonance-enhanced two-colour two-photon excitation scheme^39,46,48,49^ depicted in Fig. 2. Counter-propagating radiation from a pair of Nd:YAG pumped pulsed dye lasers, with full-width-at-half-maximum (FWHM) spectral widths of 0.17 cm^−1^, was used for photoexcitation. The frequency-tripled output of the first laser was set to *υ*_1_ = 44193.99 cm^−1^ (≡226.275 nm), a pulse energy of ∼10 μJ, and linearly polarised in the *z* dimension to drive the A ^2^Σ^+^ ← X ^2^Π_1/2_ transition. The frequency-doubled output of the second laser was tuned over the wavenumber range from *υ*_2_ = 30 420 cm^−1^ to 30 500 cm^−1^ (≡328.7 nm to 327.8 nm), had a pulse energy of ∼1.6 mJ and was linearly polarised in the *y* dimension to access *n*p(0) and *n*f(2) Rydberg states from the intermediate A ^2^Σ^+^ state. The fundamental wave numbers of the dye lasers were monitored using a fibre-coupled wavelength meter with an absolute accuracy of ±0.005 cm^−1^. At the time of photoexcitation, gated time-dependent electric potentials could be applied to electrode E1 to enhance population transfer to long-lived ℓ-mixed hydrogenic Rydberg–Stark states.

After photoexcitation the excited Rydberg molecules travelled for 5.3 μs (∼4 mm) before being loaded into the travelling electric traps of a chip-based transmission-line Rydberg–Stark decelerator.^47^ The decelerator was operated by applying a set of 5 sinusoidally oscillating electric potentials of amplitude *V*_0_ to the segmented centre-conductor electrodes (see Fig. 1), and a constant offset potential of −*V*_0_/2 to electrode E2. Molecules confined in the travelling traps were decelerated from 795 ms^−1^ to zero mean velocity in the laboratory-fixed frame of reference, over a distance of ∼100 mm, and in a time of ∼250 μs. In each cycle of the experiment the bunch of molecules excited was loaded into a single travelling trap. The curvature of the decelerator in the *xz*-plane minimised effects of collisions of decelerated and trapped molecules with the trailing component of the molecular beam.^34^ To minimise blackbody radiation induced transitions during deceleration and trapping the decelerator was enclosed in a heat shield and cooled to 30 K.

The trapped molecules were detected *in situ* within the decelerator structure by pulsed electric field ionisation (PFI) upon the application of a pulsed potential of +500 V to the side electrodes [see Fig. 1(a)]. The resulting NO^+^ ions were accelerated through the 2-mm-diameter aperture A1 in electrode E2, and collected on a microchannel plate (MCP) detector. Measurements were performed with rapidly switched PFI potentials (rise time ∼50 ns), to detect all Rydberg molecules present in the detection region above A1. To obtain information on the populations of the Rydberg states at the time of ionisation, slowly-rising pulsed potentials could be applied. These had a time-dependence of the form *V*_Ramp_(*t*) = −*V*_max_[1 − exp(−*t*/*τ*)], with the time constant *τ* = 4.4 μs. When this slowly rising pulsed potential was applied, ionised electrons were accelerated through aperture A1 to the MCP. The shorter flight time of the electrons to the MCP detector (typically <10 ns), than the NO^+^ ions (typically ∼1 μs), permitted the most direct mapping of the detection time of the electrons to the potential applied at the time of ionisation.^50^

The alignment of the apparatus was optimised by performing time-of-flight (TOF) measurements with the decelerator off, and PFI above the on-axis aperture A2 in electrode E2. Decelerated and trapped molecules were detected by PFI above the off-axis aperture A1. PFI at times beyond those for which the trap in the decelerator in which the molecules were confined was brought to rest, allowed studies of the rate of decay of the molecules.

## 3 Stark effect in Rydberg states of NO

To aid the interpretation of the results of the experiments, calculations of the energy-level structure and lifetimes of the Rydberg states in NO in the presence of externally applied electric fields were performed. The methods used in these calculations follow those reported in ref. 44, 46, 51 and 52, and involved determining the eigenvalues and eigenvectors of the Hamiltonian matrix describing the interaction of the molecule with an external electric field.

Although the *J*′′ = 1/2 rotational level of the X ^2^Π_1/2_ ground electronic state in NO can be described purely in Hund's angular momentum coupling case (a), excited rotational levels, including the X ^2^Π_1/2_(*v*′′ = 0, *J*′′ = 3/2) state used in the laser photoexcitation scheme implemented here, are intermediate cases between Hund's case (a) and Hund's case (b).^53,54^ On the other hand, the high Rydberg states of NO, prepared here for deceleration and electrostatic trapping, are most readily described in Hund's case (d).^44^ Calculations of the Stark effect in these Rydberg states were therefore performed in the |*n*ℓ*N*^+^*NM*_*N*_〉 Hund's-case-(d) basis where *n* and ℓ are the principal and orbital angular momentum quantum numbers of the Rydberg electron, respectively, *N*^+^ is the rotational quantum number of the NO^+^ ion core, *N* is the total angular momentum quantum number excluding spin 
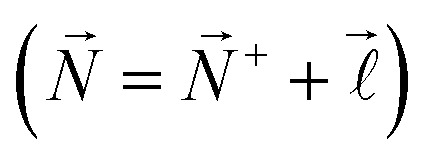
, and *M*_*N*_ is the projection of *N⃑* onto the *z*-axis in the laboratory frame of reference. Here this *z*-axis is defined by the local external electric field vector at the position of the molecule. In this basis, the total Hamiltonian can be expressed as,

1 *H*^(d)^_tot_ = *H*^(d)^_0_ + *H*^(d)^_Stark_,

where *H*^(d)^_0_ and *H*^(d)^_Stark_ are the field-free and Stark Hamiltonians, respectively. *H*^(d)^_Stark_ contains all contributions arising from the presence of the external electric field.

### 3.1 Field-free Hamiltonian

For Rydberg states in NO, the field-free Hamiltonian, *H*^(d)^_0_ in eqn (1), contains diagonal contributions that represent the energies of the unperturbed Hund's-case-(d) eigenstates. These were calculated using the Rydberg formula. Off-diagonal contributions to *H*^(d)^_0_ arise from the interactions of the Rydberg electron with the electric dipole moment, electric quadrupole moment, and electric polarisability of the NO^+^ ion core.^55–57^ They also contain contributions from the multi-electron character of NO^+^ which causes sσ–dσ configuration mixing^44,52^ (*σ* represents the *λ* = 0 projection of the Rydberg electron orbital angular momentum vector onto the internuclear axis).

The general approach used here to construct the matrix *H*^(d)^_0_ follows that presented by Goodgame *et al.*,^44^ which itself is based on the work of Fredin *et al.*^52^ on the determination of off-diagonal elements of the field-free Hamiltonian from a first order approximation to multichannel quantum defect theory (MQDT). Off diagonal matrix elements arising from the interaction of the Rydberg electron with the electric dipole moment of the NO^+^ ion were calculated using the method described by Bixon and Jortner.^43^

To construct the Hamiltonian, *H*^(d)^_0_, it was separated into a diagonal part, *H*^(d)^_diag_, a part that accounts for the interaction of the Rydberg electron with the static electric dipole moment of the NO^+^ ion core, *H*^(d)^_dip_, and a part that accounts for the combination of the interaction of the Rydberg electron with the electric quadrupole moment and higher order multipole moments of NO^+^, and the multi-electron character of the molecule, *H*^(d)^_multi_, *i.e.*,

2 *H*^(d)^_0_ = *H*^(d)^_diag_ + *H*^(d)^_dip_ + *H*^(d)^_multi_.

The Hamiltonian *H*^(d)^_diag_ is diagonal in the |*n*ℓ*N*^+^*NM*_*N*_〉 basis with matrix elements given by,3
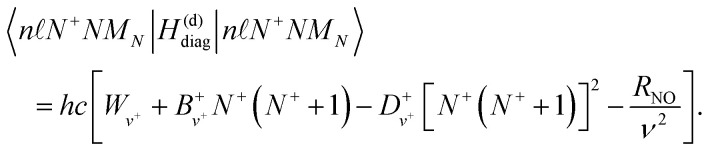
Here, *W*_*v*^+^_ is the ionisation wavenumber of the Rydberg series converging to the ground electronic state of the NO^+^ cation with vibrational quantum number *v*^+^. 
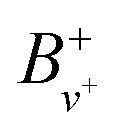
 and 
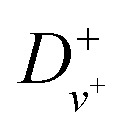
 are the rotational and centrifugal distortion constants for NO^+^. The Rydberg constant adjusted for the reduced mass of NO is *R*_NO_ = 109735.31 cm^−1^, and 
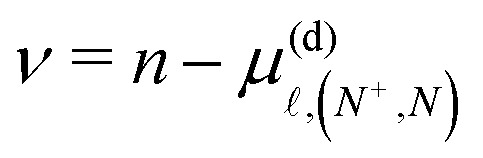
 represents the effective principal quantum number calculated using the Hund's-case-(d) quantum defects 
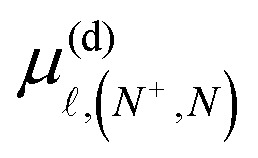
. For *v*^+^ = 0, the rotational constant is *B*^+^_0_ = 1.987825 cm^−1^, and the centrifugal distortion constant is *D*^+^_0_ = 5.64 × 10^−6^ cm^−1^.^46^

The elements of the Hamiltonian matrix *H*^(d)^_dip_ are given by,^43,56,57^4
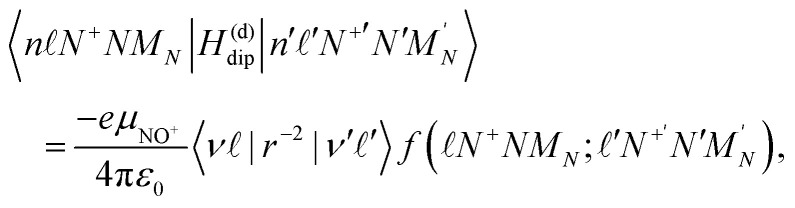
where *e* is the electron charge, *ε*_0_ is the vacuum permittivity, *μ*_NO^+^_ is the electric dipole moment of NO^+^ in a coordinate system with its origin at the centre of mass of the molecule, and 〈*ν*ℓ|*r*^−2^|*ν*′ℓ′〉 is a radial integral. The angular integral,5
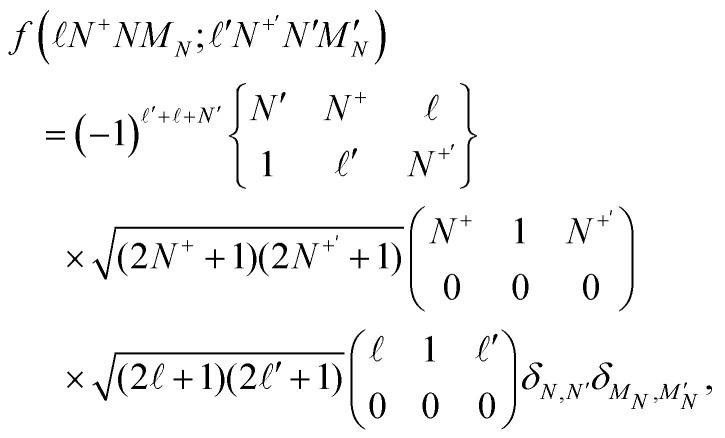
where the terms in the ( ) and { } parentheses represent Wigner 3J and 6J symbols, respectively, and *δ*_*N*,*N*′_ and 
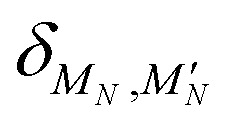
 are Kronecker delta functions. *H*^(d)^_dip_ leads to interactions between Hund's-case-(d) basis states for which,

6 Δℓ = ±1; Δ*N*^+^ = ±1; Δ*N*= 0; Δ*M*_*N*_ = 0.

The radial integral, in eqn (4), was calculated using the Gilbert-Child^58^ near-threshold approximation with a finite quantum defect such that,7
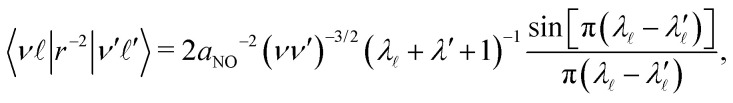
where *a*_NO_ is the Bohr radius adjusted for the reduced mass of NO, and 
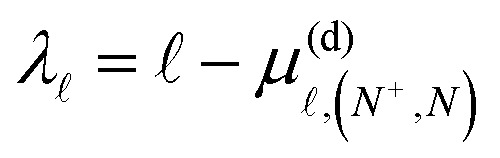
. This charge-dipole interaction is strongest between low-ℓ states, *i.e.*, states with non-zero quantum defects. In these calculations, a static electric dipole moment of NO^+^ of *μ*_NO^+^_ ≃ 0.4 D was used. This was based on the value reported from calculations, at the equilibrium internuclear separation, for the X^+ 1^Σ^+^ state of the NO^+^ ion.^59–62^

The elements of the Hamiltonian matrix *H*^(d)^_multi_ can be expressed as,^44^8
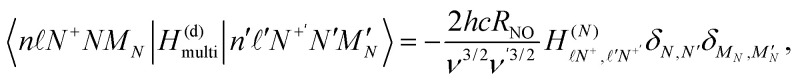
with9
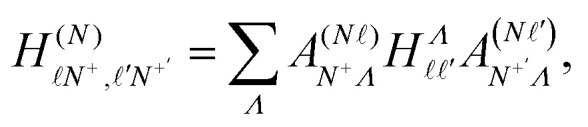
where 
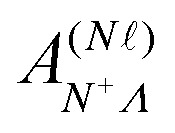
 represents the Hund's-case-(b) to Hund's-case-(d) frame transformation, and 
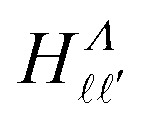
 are Hund's-case-(b) matrix elements.

For Rydberg states converging to the ^1^Σ^+^ state of NO^+^ with *Λ*^+^ = 0 (*Λ*^+^ is the projection of the total electron orbital angular momentum vector of the NO^+^ cation onto the internuclear axis), the projection of the total orbital angular momentum vector in the neutral NO molecule onto the internuclear axis is *Λ* = *λ*.^52^ With this in mind 
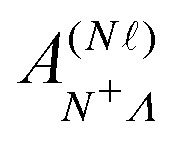
 can be expressed as,^37,44,63,64^10
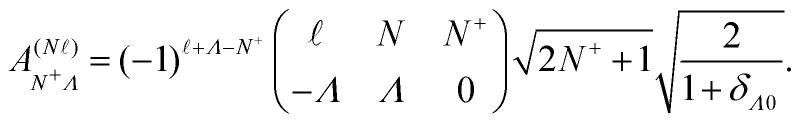
Note that the Rydberg-electron spin and spin projection are common to Hund's-case-(b) and Hund's-case-(d) so are not considered in these transformations.^63^

In NO, the configuration mixing of importance in the work described here occurs predominantly between the sσ (ℓ = 0, *Λ* = 0) and dσ (ℓ = 2, *Λ* = 0) Hund's-case-(b) basis states, and therefore results in ℓ-mixing of the Rydberg eigenstates. In Hund's-case-(b), the corresponding matrix elements, that account for these effects of configuration mixing, are,^44,52^11
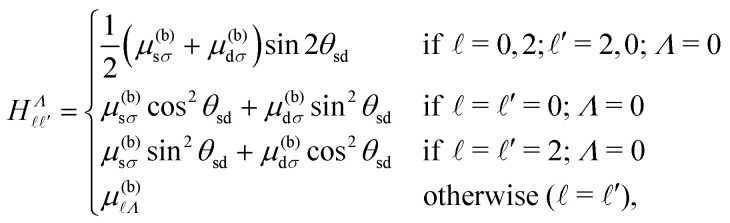
where *θ*_sd_ = −38.7° is the sσ–dσ mixing angle,^52^ and *μ*^(b)^_ℓ*Λ*_ are the Hund's-case-(b) quantum defects. Taking this into account, the complete sets of selection rules for the intramolecular interactions accounted for in *H*^(d)^_multi_ are,

12 Δℓ = 0, ±2; Δ*N*^+^ = ±2; Δ*N* = 0; Δ*M*_*N*_ = 0,

where the Δℓ = ±2 interactions only occur between basis states with ℓ = 0 and ℓ′ = 2, and *vice versa*. The Hund's-case-(b) quantum defects, *μ*^(b)^_ℓ*Λ*_, used in the calculations are listed in Table 1. To obtain the Hund's-case-(d) quantum defects, 
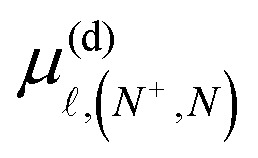
, required in the determination of *ν* in eqn (3), (4), and (8), a frame transformation was performed such that,13
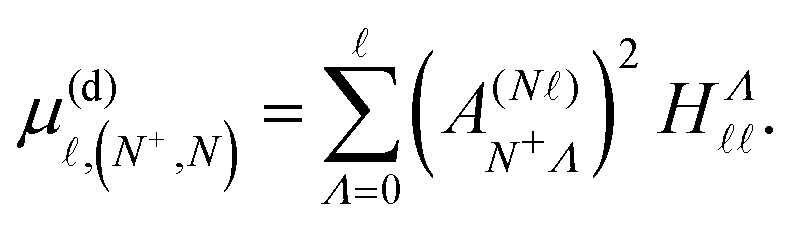


**Table 1:** Hund's-case-(b) quantum defects, *μ*^(b)^_ℓ*Λ*_, for ℓ ≤ 3 from ref. 46

		*Λ*			
		0	1	2	3
ℓ	0	0.210			
	1	0.7038	0.7410		
	2	0.050	−0.053	0.089	
	3	0.0182	0.0172	0.00128	0.0057

### 3.2 Stark Hamiltonian

In an electric field *F⃑* = (0, 0, *F*_*z*_), the Hund's-case-(d) Hamiltonian in eqn (2) can be extended to include contributions from the Stark effect. The resulting perturbation, *H*^(d)^_Stark_ = *eF*_*z*_*z*, has matrix elements given by,^37,65^14
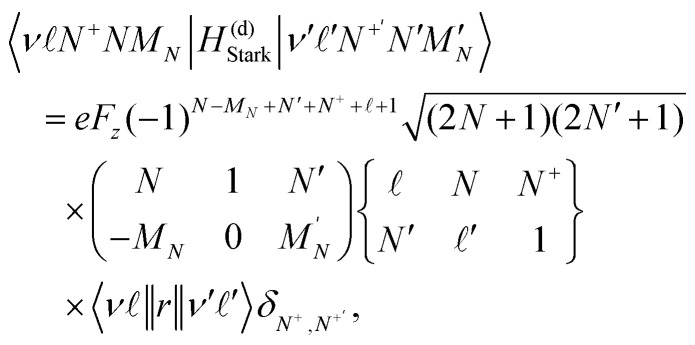
where the integral,15
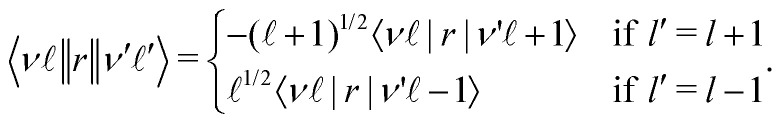
This perturbation leads to non-zero off diagonal couplings between states, within the same rotational series, for which Δℓ = ±1, *i.e.*, between states for which,

16 Δℓ = ±1; Δ*N*^+^ = 0; Δ*N* = 0, ±1 (not 0 → 0); Δ*M*_*N*_ = 0.

The radial integrals 〈*ν*ℓ|*r*|*ν*′ℓ′〉 on the right-hand side of eqn (15) were evaluated using the Numerov method^66^ with a pure Coulomb potential. Stepwise integration was performed inwards from large *r* to either the inner classical turning point of the Rydberg electron orbit, or the polarisability radius, *r*_*α*_, of the NO^+^ ion core whichever was encountered first. The polarisability radius, *r*_*α*_, was taken to be *r*_*α*_ ≃ (*α*_NO^+^_)^1/3^,^66^ where *α*_NO^+^_ ≃ 8 a.u. is the electric dipole polarisability of the NO^+^ cation.^67,68^

### 3.3 Lifetime calculations

Using the eigenvectors obtained following diagonalisation of *H*^(d)^_tot_, the lifetimes of the Rydberg states in NO in the presence of an electric field could be determined. From the rates of decay by fluorescence, *γ*_fl(*k*)_, and dissociation, *γ*_dis(*k*)_, of each basis state, denoted |*k*〉 = |*n*_*k*_ℓ_*k*_*N*^+^_*k*_*N*_*k*_*M*_*N*_*k*__〉, the total decay rate of an eigenstate |*i*〉 of *H*^(d)^_tot_ is,17
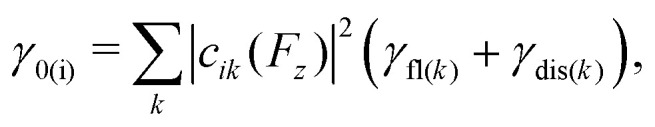
where *c*_*ik*_(*F*_*z*_) = 〈*k*|*i*〉^(*F*_*z*_)^ are the coefficients of the corresponding eigenvector in the electric field *F*_*z*_.

The fluorescence rates *γ*_fl(*k*)_ = 1/*τ*_fl(*k*)_ of the basis states were calculated by considering the zero-field lifetimes, *τ*_*n*ℓ_, of the hydrogenic Rydberg states averaged over all polarisations. The effect of ℓ-mixing was then accounted for through the dependence of *c*_*ik*_(*F*_*z*_) on the field strength. The fluorescence lifetimes can be expressed as,18
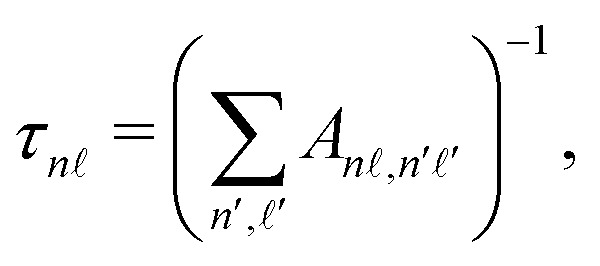
where *A*_*nℓ*,*n*′*ℓ*′_ are the Einstein *A* coefficients, and the primes denote states energetically below the |*n*, ℓ〉 state. The values of *A*_*nℓ*,*n*′*ℓ*′_ are given by,^69^19
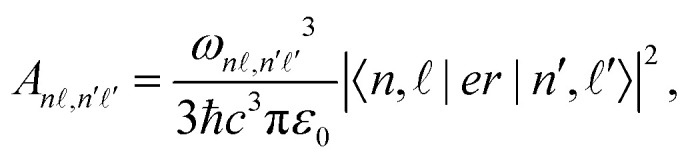
with *ω*_*n*ℓ,*n*′ℓ′_ = (*E*_*n*ℓ_ − *E*_*n*′ℓ′_)/*ħ*, and *ħ* the reduced Planck constant.

The dissociation rates, *γ*_dis(*k*)_, required in eqn (17) are dependent upon *ν*_*k*_ and ℓ_*k*_. This dependence can be expressed as,^43^20
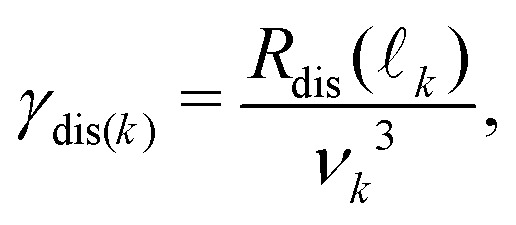
where *R*_dis_(ℓ_*k*_) is the dissociation rate parameter for a basis state in the *v*^+^ = 0 series with ℓ = ℓ_*k*_. The dissociation rate parameters for Rydberg states in the *v*^+^ = 0 series that are available in the literature for values of ℓ ≤ 4, and were used in the calculations described here, are presented in Table 2. For higher values of ℓ, *R*_dis_(ℓ) was assumed to be zero.

**Table 2:** Dissociation rate parameters, *R*_dis_(ℓ), for *v*^+^ = 0 states in NO (see text for details)

ℓ	*R*_dis_(ℓ)(s^−1^)	Origin
0	9.42 × 10^13^	a
1	3.03 × 10^14^	b
2	1.88 × 10^14^	a
3	8.10 × 10^12^	b
4	1.60 × 10^11^	c

a Arbitrarily partitioned in ref. 43 from [*R*_dis_(0) + *R*_dis_(2)]/2π*c* ≃ 1500 cm^−1^.

b From ref. 41 and 43.

c From ref. 51 and 70.

**Fig. 3:**
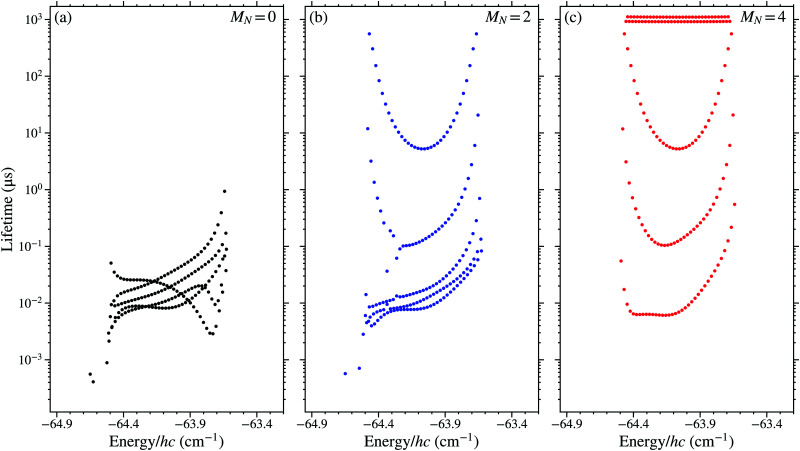
Calculated lifetimes of Rydberg–Stark states in NO with *n* = 38, *N*^+^ = 2, and *v*^+^ = 0 for (a) *M*_*N*_ = 0, (b) *M*_*N*_ = 2, and (c) *M*_*N*_ = 4. The wave numbers on the horizontal axis are shown relative to the *v*^+^ = 0, *N*^+^ = 0 series limit. All calculations were performed for an electric field of 5 V cm^−1^.

The calculated lifetimes, *τ*_0(*i*)_ = 1/*γ*_0(*i*)_, of the *n* = 38, *N*^+^ = 2 Rydberg states in the *v*^+^ = 0 series with *M*_*N*_ = 0, 2, and 4 are shown in Fig. 3(a), (b), and (c), respectively. In each panel, the 2*N*^+^ + 1 = 5 Stark manifolds can be seen. The results of all calculations shown in this figure were obtained for an electric field of 5 V cm^−1^. In this field sufficient ℓ-mixing has occurred for the calculated lifetimes to provide a reasonable representation of those of the same states in higher fields, while Stark states with different values of *n* remain clearly distinguishable allowing for ease of interpretation of the results. For low values of |*M*_*N*_|, *i.e.*, |*M*_*N*_| ≤ 2, dissociation represents the dominant contribution to the decay of the excited states and the lifetimes of the states in the different Stark manifolds overlap. For larger values of |*M*_*N*_|, dissociation plays a less important role, and the manifolds of states decay predominantly by fluorescence and therefore have distinguishable lifetimes.

The high dissociation decay rates of the low-ℓ (ℓ ≤ 3) basis states, which are between ∼10^8^ s^−1^ and ∼5 × 10^9^ s^−1^ for *n* = 38, dominate over the fluorescence decay rates (∼10^4^ s^−1^ for *n* = 38) of these states. On the other hand, for high-ℓ basis states (ℓ ≥ 4) the total decay rates are smaller, with fluorescence rates ranging from ∼10^2^ s^−1^ to ∼10^4^ s^−1^ and dissociation rates <3 × 10^6^ s^−1^. Because of the significant reduction in the dissociation rates as the value of ℓ increases, only the dissociation of basis states with ℓ ≤ 4 were considered in calculations. With this in mind, mixed states, with some high-ℓ character and some low-ℓ character, can have significantly reduced lifetimes compared to those of the higher-ℓ states in zero-electric-field. This can be seen in Fig. 3(a), where all possible ℓ values are present for *M*_*N*_ = 0, with significant low-ℓ character mixed into all Stark states. The lifetimes of states with *M*_*N*_ = 0 are all <1 μs, *i.e.*, they decay predominately by dissociation even in weak electric fields.

When |*M*_*N*_| > 0 not all low-ℓ basis states contribute to the ℓ-mixed Stark states. However individual low-ℓ states are not absent from all Stark manifolds with the same value of |*M*_*N*_|. Contributions from basis states with particular values of *N*^+^ and ℓ reduce when |*M*_*N*_| > |*N*^+^ − ℓ|, and are completely absent when |*M*_*N*_| > *N*^+^ + ℓ. Therefore, in the example case with *N*^+^ = 2, to prevent ℓ ≤ 3 basis states mixing into at least one of the Stark manifolds populated, it is necessary for |*M*_*N*_| to be greater than or equal to 2. The effect this has on the lifetimes can be seen in Fig. 3(b and c). When *M*_*N*_ = 2 [Fig. 3(b)], only one of the *N*^+^ = 2 Stark manifolds has no ℓ ≤ 3 character. This manifold is easily identifiable as that with lifetimes between ∼10 μs and ∼1 ms. The shorter lifetimes towards the centre of this manifold reflect the presence of an ℓ = 4 component which still causes some dissociative decay.

For the case in which *M*_*N*_ = 4 [Fig. 3(c)], the Stark manifolds are clearly distinguishable by the lifetimes of their component states. In the manifold with the shortest lifetimes (∼10 ns) the lowest ℓ basis states present are those for which ℓ = 2. Whereas, in the manifold with the longest lifetimes (∼1 ms) ℓ ≥ 6. In between these extreme cases the lowest ℓ basis state present lies between these values. In these data it is therefore possible to distinguish between manifolds of states that decay predominantly by dissociation and contain low-ℓ (ℓ ≤ 3) character, and those with only high-ℓ (ℓ ≥ 4) character for which decay by fluorescence dominates. The asymmetry in the lifetimes of the states of the Stark manifolds with ℓ ≤ 3 character in Fig. 3, arises because the low-ℓ states are not degenerate with those of higher ℓ in zero-field.

### 3.4 Considerations for the implementation of Rydberg–Stark deceleration

**Fig. 4:**
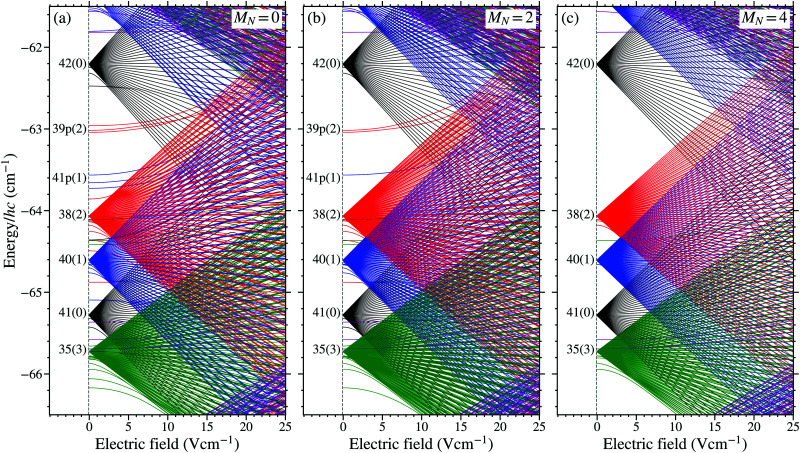
Calculated Stark maps for *v*^+^ = 0 Rydberg states in NO with (a) *M*_*N*_ = 0, (b) *M*_*N*_ = 2, and (c) *M*_*N*_ = 4. The wave numbers on the vertical axis are shown relative to the *v*^+^ = 0, *N*^+^ = 0 series limit.

An example of a Stark map calculated by diagonalising *H*^(d)^_tot_, and encompassing the wavenumber region around the field-free 38(2) state, is displayed in Fig. 4. The energy-level structure for states with *M*_*N*_ = 0, 2, and 4 is displayed in Fig. 4(a), (b), and (c), respectively. The quantum numbers used to label the individual states on the left-hand side of each panel in this figure indicate the dominant character in zero electric field. In this labelling scheme, a distinction is made between non-degenerate low-ℓ (ℓ ≤ 3) states, for which the dominant ℓ character can be determined in weak fields, and high-ℓ (ℓ ≥ 4) degenerate states.

For Rydberg–Stark deceleration and electrostatic trapping,^18,71^ ℓ-mixed Stark states with positive Stark energy shifts, *i.e.*, low-field-seeking (LFS) states, are required. These states must have lifetimes greater than ∼10 μs, and evolve diabatically through any avoided crossings in the Stark map in the time-dependent electric fields used for deceleration and trapping.^18^ These requirements demand that contributions from short-lived, non-degenerate low-ℓ basis states are minimised. This can be achieved, as seen, *e.g.*, from the data in Fig. 3, by populating states with high values of |*M*_*N*_|, *i.e.*, |*M*_*N*_| ≥ 3.^18^

The effect of increasing the value of |*M*_*N*_|, on the Stark energy level structure of the Rydberg states, can be seen in Fig. 4. For *M*_*N*_ = 0 [Fig. 4(a)], basis states with all values of ℓ are present in the Stark map. Contributions from the low-ℓ states in this case lead to short lifetimes and large avoided crossings for LFS states. For larger values of |*M*_*N*_|, *e.g.*, |*M*_*N*_| = 4 in Fig. 4(c), basis states with ℓ < |*M*_*N*_| − *N*^+^ are absent. The absence of these low-ℓ states results in longer lifetimes and smaller avoided crossing throughout the Stark map.

Rydberg states with |*M*_*N*_| ≥ 3 can be selectively populated by resonance-enhanced multi-photon excitation with circularly polarised laser radiation as achieved, *e.g.*, in the case of H_2_.^18^ They can also be populated by *M*_*N*_-mixing in collisions and in the presence of time-varying electric fields.^72,73^ In this latter case, deceleration and electrostatic trapping acts to filter the long-lived high-|*M*_*N*_| components of an ensemble of excited molecules. This filtering of long-lived high-|*M*_*N*_| states occurs because of the finite time required for deceleration, which is only effective for molecules in long-lived Rydberg states, and the requirement to undergo diabatic traversal of avoided crossings in the deceleration fields, that only occurs with high probability for high values of |*M*_*N*_|.

The two-colour two-photon laser photoexcitation scheme used in the work reported here led to the population of long-lived hydrogenic Rydberg–Stark states in NO through collisions and the effects of time-varying electric fields, as demonstrated previously by state-selective electric field ionisation measurements.^39^ Although direct measurements of the lifetimes of the states excited were not made previously, the geometry of the apparatus meant that lifetimes in excess of 10 μs were required for the excited molecules to be detected. This observation, in conjunction with the calculated lifetimes in Fig. 3, suggest that hydrogenic Rydberg–Stark states without any low-ℓ (ℓ ≤ 3) character, *i.e.*, states with |*M*_*N*_| ≥ 3, were therefore populated.

## 4 Results

The methods of Rydberg–Stark deceleration and electrostatic trapping of NO molecules in long-lived Rydberg–Stark states that were employed in the work reported here, have allowed detailed studies of the decay of these excited states on time scales up to 1 ms. Experiments were performed to investigate: (i) the range of values of *n* and *N*^+^ of the Rydberg states that could be efficiently decelerated and electrostatically trapped in the apparatus, (ii) the effects of transitions induced by blackbody radiation on the Rydberg state populations in the trap, and (iii) the range of trapping times over which quantum-state-dependent decay rates of the molecules from the trap could be reliably determined. The results of this work, to characterise and validate the measurement methodology are described in the following in Section 4.1. Measurements of quantum-state-dependent decay rates of molecules from the trap are then presented in Section 4.2. The interpretation of these results, in terms of effects of rotational and vibrational channel interactions, are discussed in Sections 4.4 and 4.3, respectively.

### 4.1 Trap decay measurements

**Fig. 5:**
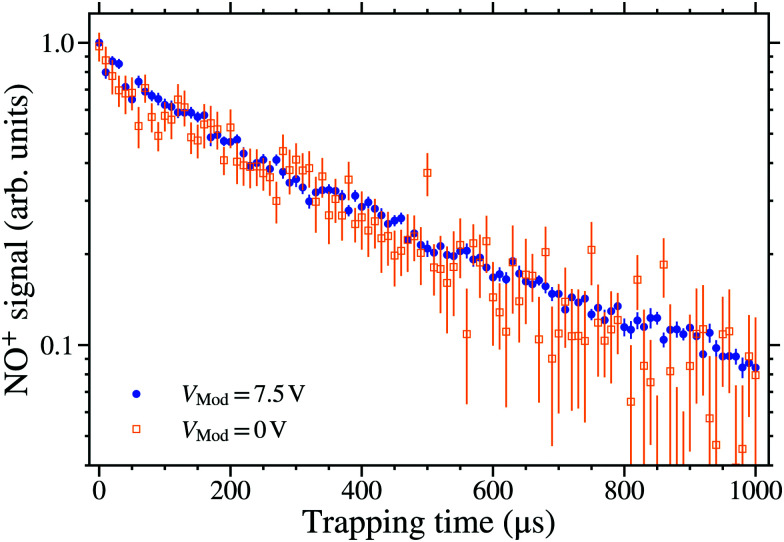
Decay of trapped Rydberg NO molecules, photoexcited on the 38f(2) resonance at *υ*_2_ = 30458.37 cm^−1^, with (*V*_Mod_ = 7.5 V) and without (*V*_Mod_ = 0 V) a 50 MHz gated sinusoidal potential applied to the electrode E1 at the time of photoexcitation (see text for details). For both measurements *V*_0_ = 149 V and the data were normalised to the NO^+^ signal recorded at time *t*_trap_ = 0 μs.

To measure rates of decay of electrostatically trapped Rydberg NO molecules, laser photoexcitation and Rydberg–Stark deceleration were performed as described in Section 2. When the traps in which the molecules were confined were brought to rest, at time *t*_trap_ = 0 μs in the following, *in situ* detection by PFI was performed for times up to *t*_trap_ = 1 ms. Examples of the results of such trap decay measurements, for molecules photoexcited on the 38f(2) resonance (*υ*_2_ = 30458.37 cm^−1^), can be seen in Fig. 5. There are two main components to the decay dynamics observed in these data: (i) at early trapping times a fast reduction in signal is observed, reflecting the decay of the Rydberg states predominantly populated upon photoexcitation, and (ii) at later times this reduction slows down reflecting the presence of some molecules in states with longer lifetimes that remain in the trap at late times.^47,74^

The data indicated by the open squares in Fig. 5 represent measurements made upon laser photoexcitation in nominally zero electric field, *V*_Mod_ = 0 V. However, because the mechanism by which long-lived Rydberg–Stark states are populated close to the time of laser photoexcitation depends on *M*_*N*_-mixing that occurs in time-varying electric fields, this can be enhanced by applying a rapidly-varying field in the excitation region when the laser radiation is present. The effect of this sinusoidal field modulation, generated by applying a gated sinusoidally oscillating electric potential to electrode E1 (see Fig. 1) with an amplitude of *V*_Mod_ = 7.5 V and a frequency of 50 MHz for a time of 50 ns, can be seen by comparing the data recorded with (blue circles) and without (orange open squares) this modulation. The rapid modulation of the field in the excitation region resulted in a factor of ∼30 increase in the NO^+^ signal detected by PFI, compared to photoexcitation performed with *V*_Mod_ = 0 V, and hence an increase in the number of molecules in long-lived Rydberg–Stark states that were trapped. The corresponding increase in signal meant that upon the application of this field modulation, molecules could be observed in the trap for times up to 1 ms. Without this field modulation the NO^+^ signal reduced to the level of the background noise after a trapping time of ∼500 μs. The introduction of this field modulation does not change the measured decay time constants from the trap at early times. An exponential function fitted to both sets of data in Fig. 5, for trapping times between 0 and 500 μs, resulted in decay time constants of 366 ± 6 μs and 377 ± 21 μs for the cases with and without the modulation, respectively. From this it is concluded that rapid electric field modulation close to the time of laser photoexcitation leads to the population of Rydberg–Stark states with similar characteristics to those populated as a result of effects of electric field noise, and the presence of ions. In the following, all measurements were therefore performed with the 50 MHz sinusoidal potential, of amplitude *V*_Mod_ = 7.5 V, applied at the time of photoexcitation.

**Fig. 6:**
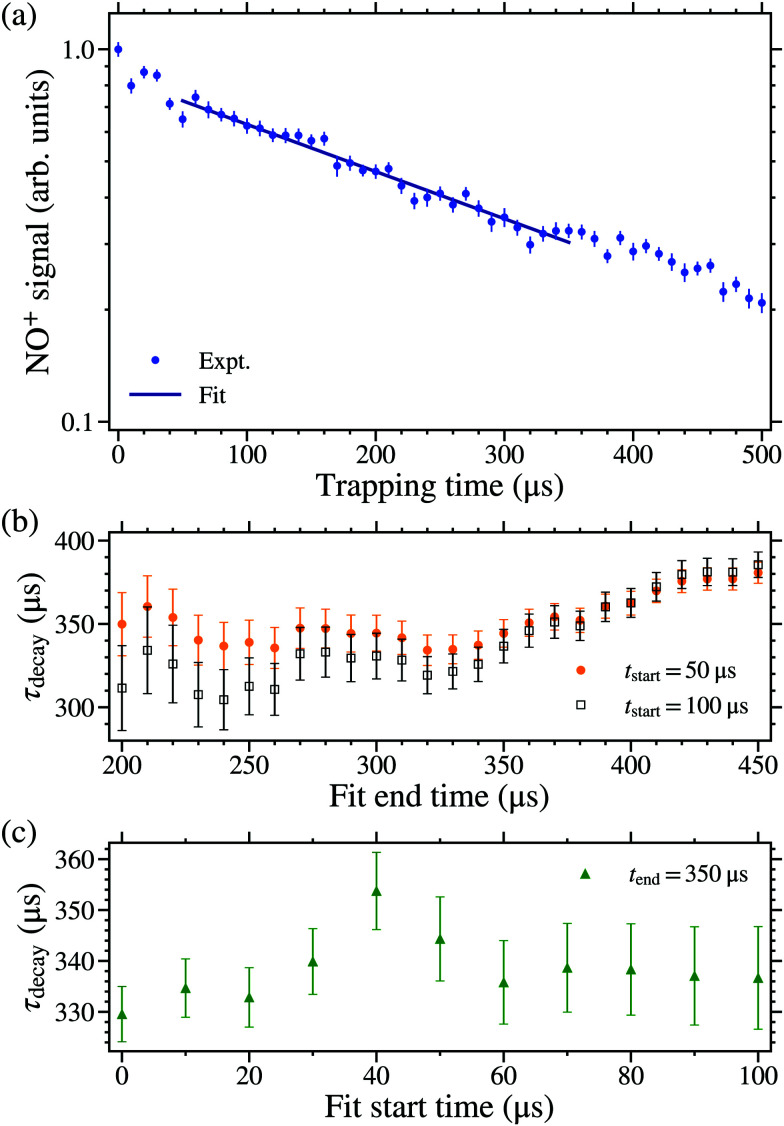
(a) Measured decay of Rydberg NO molecules, excited on the 38f(2) resonance, from the electrostatic trap, with an example of a fitted exponential function encompassing the times from *t*_trap_ = 50 μs to 350 μs. (b) The variation in *τ*_decay_ associated with fitting between 50 μs (orange circles) or 100 μs (black open-squares) and the fit end times indicated on the horizontal axis. (c) The variation in *τ*_decay_ associated with fitting between the fit start times indicated on the horizontal axis, and 350 μs.

To measure quantum-state-dependent decay rates of the trapped molecules single exponential functions were fit to the experimental data, such as that in Fig. 5, over a range of early trapping times to obtain the decay time constant, *τ*_decay_. To validate the fitting procedure, and minimise spurious effects arising from the motion of the molecules in the trap, the range of trapping times over which the fit was performed was tested to ensure the stability of values of *τ*_decay_ obtained. This was done for molecules excited on the 38f(2) resonance shown in Fig. 6(a). In Fig. 6(b) the variation in the values of *τ*_decay_ obtained for fixed fit start times of 50 μs or 100 μs, and a range of fit end times can be seen. For end times up to ∼350 μs the value of *τ*_decay_ remains constant within the the experimental uncertainties. However at later end times the value of *τ*_decay_ gradually increases as the experimental data deviates from a single exponential function. This change in the decay time constant at later trapping times reflects the fact that a range of Rydberg–Stark states were populated close to the time of photoexcitation.^74^ Molecules in shorter lived states decay more quickly from the trap, and therefore at later times only molecules in states with longer lifetimes remain. To minimise the effects of these decay dynamics on the values of *τ*_decay_, the fit end time was chosen to be 350 μs for all measurements described below.

Fig. 6(c) shows the values of *τ*_decay_ obtained for a range of fit start times. For trapping times ≤50 μs the value of *τ*_decay_ varied with the fit start time. This behaviour is a consequence of the motion of the molecules in the trap after it is brought to rest.^32,47^ During deceleration the molecules are predominantly located ahead of the electric field minima of the travelling traps.^30^ After a trap is stopped the molecules oscillate with a period of ∼50 μs, as they evolve to fill it. From these considerations, the start time of the fitting process was therefore set to 50 μs.

**Fig. 7:**
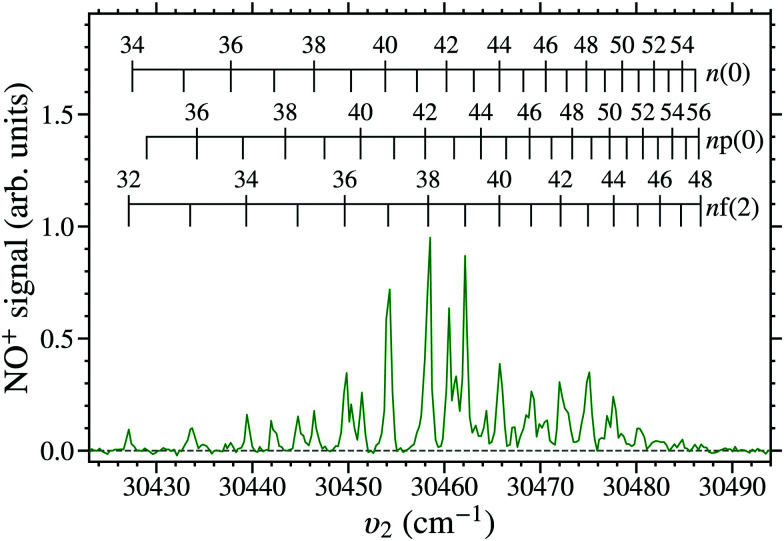
Laser photoexcitation spectrum of long-lived Rydberg states in NO recorded after deceleration and trapping for *t*_trap_ = 350 μs, with *υ*_1_ = 44193.99 cm^−1^, and *V*_0_ = 149 V.

To ascertain the range of Rydberg states suitable for decelerating and electrostatically trapping, a laser photoexcitation spectrum, shown in Fig. 7, was recorded with detection after a trapping time of *t*_trap_ = 350 μs. The spectral intensities of each of the features in this spectrum reflect the combined contributions from the efficiency with which long-lived LFS hydrogenic Rydberg–Stark states were populated, the deceleration and trap loading efficiencies, and the excited-state decay rates. The reduced spectral intensities at lower values of *n* reflect the *n*^2^ dependence of the maximal electric dipole moments of the Rydberg states, that reduce the deceleration and trapping efficiency for lower *n* states. The spectral intensities in Fig. 7 also reduce for high values of *n*. This is a consequence of the lower threshold for electric field ionisation during deceleration and trap loading, and the lifetimes of the states.^47^

The deceleration and trapping efficiency of the NO molecules depends on effects of intramolecular interactions between the optically accessible *n*p(0) or *n*f(2) states, and nearby ℓ-mixed hydrogenic Stark states. The resonances associated with transitions to the *n*f(2) states in Fig. 7 arise because of their close proximity to, and mixing with, the *n*(2) hydrogenic Stark states.^39,47^ Spectral features that arise from mixing of the *n*p(0) states and hydrogenic Stark states are also observed. Despite the *n*p(0) states being shorter lived that the *n*f(2) states,^40,41^ the hydrogenic states populated in both cases are long lived. Two manifolds of ℓ-mixed hydrogenic Stark states are accessible upon excitation because of mixing with the *n*p(0) states. These are (i) *n*′(1) (where *n*′ ≠ *n*) hydrogenic Stark states that mix because of accidental degeneracies with *n*p(0) states as a result of intramolecular charge-dipole interactions.^39,43^ These coincide with resonances denoted *n*p(0) in Fig. 7. And (ii) *n*(0) hydrogenic Stark states that mix with the [*n* + 1]p(0) states in the electric field present close to the time of photoexcitation. These states are denoted *n*(0) in Fig. 7.

**Fig. 8:**
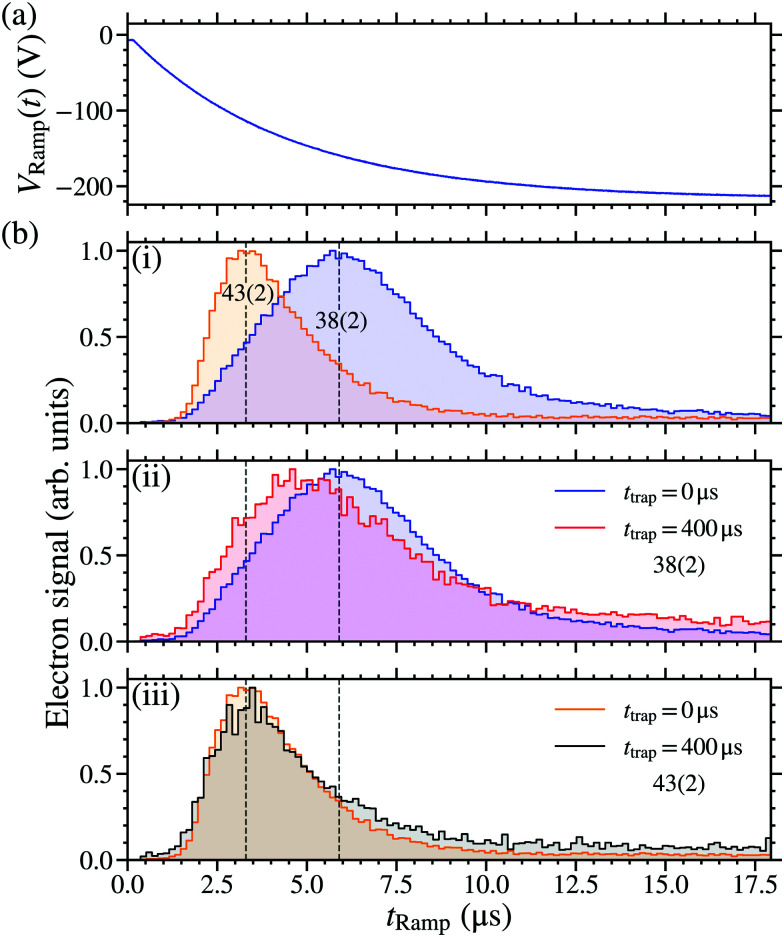
(a) The potential *V*_Ramp_(*t*) applied to the side electrodes (see Fig. 1) during state-selective PFI. (b) Measured electron signals following excitation of NO molecules on (i) the 38(2) and 43(2) resonances recorded after a trapping time of *t*_trap_ = 0 μs, (ii) the 38(2) resonance at *t*_trap_ = 0 μs and *t*_trap_ = 400 μs, and (iii) the 43(2) resonance at *t*_trap_ = 0 μs and *t*_trap_ = 400 μs.

The use of state-selective PFI to detect trapped Rydberg NO molecules allowed information on the time-evolution of the excited state populations to be determined. To achieve this PFI was performed by turning off the trapping potentials, and ∼1 μs later applying a slowly-rising ionising potential *V*_Ramp_(*t*) to the side electrodes of the decelerator. The time-dependence of *V*_Ramp_(*t*) is shown Fig. 8(a). The electron signal obtained at a trapping time of *t*_trap_ = 0 μs, for molecules excited on the 38(2) and 43(2) resonances, are shown in Fig. 8(b)-(i). From these data a distinction between the electron detection times, and hence the ionisation fields of the molecules, can be identified. The maxima in the signals recorded following excitation on the 38(2) and 43(2) resonances, occur at *t*_Ramp_ ∼ 5.9 μs [*V*_Ramp_(5.9 μs) ∼ −160 V] and ∼3.3 μs [*V*_Ramp_(3.3 μs) ∼ −110 V], respectively. Since the maximum in the electron signal associated with the 43(2) resonance occurs earlier than that associated with the 38(2) resonance, it is concluded that excitation on the 43(2) resonance leads to ionisation in smaller electric fields. This is expected from the *n*-dependence of the ionisation field and previous detailed measurements of the electric field ionisation dynamics of long-lived hydrogenic states in NO.^39^ The widths of the distributions in Fig. 8(b)-(i) reflect the combined effects of a distribution of Stark states present in the trap at the time of PFI, the inhomogeneity of the ionisation field arising as a result of the electrode geometry in the decelerator, and the motion of the molecules in this time-varying inhomogeneous electric field after the trap is switched off. The tails in the electron signals at late times in Fig. 8(b)-(i) are attributed to secondary electron generation following PFI.

The expected ratio of the ionisation fields for LFS hydrogenic Rydberg–Stark states with different values of *n* was determined from the ionisation field, *F*_ion_(*n*), of the outermost LFS Stark state,

21 *F*_ion_(*n*) = 2*F*_0_/9*n*^4^,

where *F*_0_ = 2*hcR*_NO_/*ea*_NO_. For the 43(2) and 38(2) states this ratio is *F*_ion_(43)/*F*_ion_(38) = 0.6. This value is comparable to the ratio of the values of *V*_Ramp_(*t*) at the maxima of the electron distribution for the molecules excited on the 43(2) and 38(2) resonances of *V*_Ramp_(3.3 μs)/*V*_Ramp_(5.9 μs) ∼ 0.7. It is therefore concluded that the molecules trapped in these states predominately undergoing diabatic electric field ionisation.^39^

Using this state selective PFI detection scheme, information on the evolution of the excited state populations was also obtained by comparing the electron signals recorded when *t*_trap_ = 0 μs and 400 μs. This was done for the 38(2) and 43(2) resonances with the results, scaled such that the signal maximum in each case is 1, are shown in Fig. 8(b)-(ii and iii), respectively. For molecules excited on the 43(2) resonance, no significant change in the general form of the electron TOF distribution was observed between *t*_trap_ = 0 μs and 400 μs. For molecules excited on the 38(2) resonance this is not the case. Under these conditions when *t*_trap_ = 400 μs the maximum of the electron signal occurred at *t*_Ramp_ ∼ 4.8 μs [*V*_Ramp_(4.8 μs) ∼ −140 V], *i.e.*, approximately 1.1 μs before that recorded when *t*_trap_ = 0 μs. This suggests that the molecules detected at late times in this case are in higher-*n* states. From the ratio of the corresponding values of *V*_trap_(*t*), this shift in the maximum of the electron signal is estimated to correspond to a change in *n* of ∼+2.

By performing numerical particle trajectory calculations assuming diabatic traversal of all avoided crossings between Stark states,^47^ the typical distribution of the maximum electric fields experienced by molecules when trapped was determined. The mean of this distribution was ∼150 V cm^−1^. As an estimate of the electric field ionisation rates of the trapped NO molecules in the experiments, it can be determined that for hydrogenic Rydberg–Stark states with *n* = 38, |*m*| = 4, *k* = +33 (*n* = 43, |*m*| = 4, *k* = +38), tunnel ionisation would occur at a rate of ∼10^−30^ s^−1^ (∼10^−3^ s^−1^) in this field^75,76^ (*k* = *n*_1_ − *n*_2_ is the difference in the parabolic quantum numbers *n*_1_ and *n*_2_ obtained by solving the Schrödinger equation for a pure Coulomb potential in parabolic coordinates^77^). Hence, it is concluded that electric field ionisation arising from the motion of molecules in the trap does not affect the distribution of states with these values of *n* populated over time.

In a similar vein, for hydrogenic Stark states with *n* = 38, |*m*| = 4, *k* = +33 (*n* = 43, |*m*| = 4, *k* = +38), the Δ*n* = ±1 and ±2 blackbody transitions rates at 30 K are ∼9 × 10^2^ s^−1^ and ∼4 × 10^2^ s^−1^ (∼7 × 10^2^ s^−1^ and ∼3 × 10^2^ s^−1^), respectively. At this temperature these depopulation rates scale approximately with *n*^−2^. However, because the molecules are trapped ∼2 mm above a 2-mm-diameter aperture in the heat shield surrounding the decelerator (A1 in Fig. 1), the blackbody field that the molecules interact with has a room temperature component. By considering the fractional solid angle subtended by the aperture at the minimum of the trap this corresponds to approximately 0.25, not accounting for modifications in the spectrum arising from the dimensions of the electrode structure. At 300 K the Δ*n* = ±1 and ±2 blackbody depopulation rates, for *n* = 38, |*m*| = 4, *k* = 33 (*n* = 43, |*m*| = 4, *k* = 38), are ∼1 × 10^4^ s^−1^ and ∼5 × 10^3^ s^−1^ (∼7 × 10^3^ s^−1^ and ∼3 × 10^3^ s^−1^), respectively. Therefore the total Δ*n* = ±1 and ±2 blackbody depopulation rates for these states at the position of the trap are estimated to be below 0.75*γ*_BB_(30 K) + 0.25*γ*_BB_(300 K) and hence ∼3 × 10^3^ s^−1^ and ∼1 × 10^3^ s^−1^ (∼2 × 10^3^ s^−1^ and ∼1 × 10^3^ s^−1^), respectively. These blackbody transition rates are similar for both *n* = 38 and *n* = 43. Therefore any blackbody-induced changes in the electron distribution, measured by PFI, are not expected to differ for the molecules excited to the 38(2) and 43(2) states. Consequently, the change in the *n* = 38 electron distribution with trapping time [see Fig. 8(b)-(ii)] is interpreted to reflect the initial population of the Rydberg states close to the time of photoexcitation and before deceleration and trapping. Excited states with longer lifetimes remain in the trap for longer times. Hence if an ensemble of molecules is prepared in a range of states, with different lifetimes, the measured distribution of states populated in the trap will change over time. This behaviour has been discussed previously by Seiler *et al.* in the interpretation of experiments with electrostatically trapped Rydberg H atoms.^74^ In the case of interest here, this suggests that excitation of the NO molecules on the 38f(2) resonance resulted in the population of at least two Rydberg states with different values of *n*(*N*^+^), while excitation on the 43f(2) resonance lead predominantly to the population of states with a single set of values of *n*(*N*^+^).

### 4.2 Decay rate measurements

**Fig. 9:**
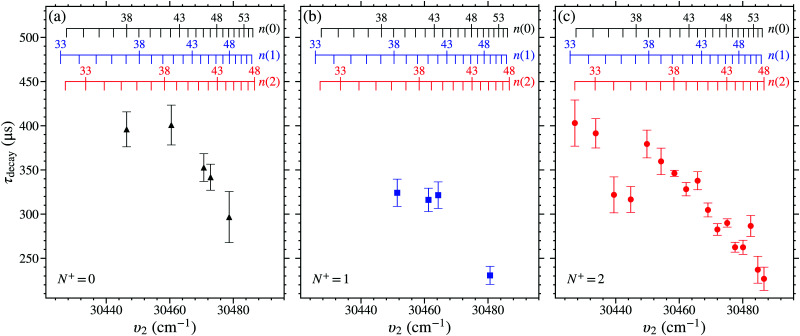
Measured values of *τ*_decay_, for *V*_0_ = 149 V, of Rydberg–Stark states in NO with (a) *N*^+^ = 0, (b) *N*^+^ = 1, and (c) *N*^+^ = 2.

Measurements of the trap decay time constants, *τ*_decay_, for NO molecules in *n*(0), *n*(1), or *n*(2) Stark states were performed for values of *n* between 32 and 50. The value of *τ*_decay_ was determined from each of these measurements by fitting a single exponential function to the experimental data between *t*_trap_ = 50 μs and 350 μs, as described in Section 4.1. The weighted mean and standard deviation of up to 6 separate measurements of *τ*_decay_ for each set of values of *n*(*N*^+^) were calculated. These values of *τ*_decay_ obtained for the *n*(0), *n*(1), and *n*(2) states, with *V*_0_ = 149 V, can be seen in Fig. 9. For reference, the decay time constants of the 38(2) and 43(2) states in this figure were *τ*_decay_ = 346.2 ± 3.4 μs and 290.0 ± 4.7 μs, respectively.

From the measured trap decay times in Fig. 9 it is seen that there is a general trend for the measured value of *τ*_decay_ to decrease with increasing values of *n*, and therefore also increasing excitation wavenumber, *υ*_2_. This behaviour occurs for states in each *N*^+^ series studied. For the *n*(0) and *n*(1) states this trend is seen for *n* ≥ 40. For the *N*^+^ = 2 series, the general reduction in the value of *τ*_decay_ with *n* begins at lower values of *n*. But for *n* = 34, 35, 40, 43, 45, and 46, deviations from this general behaviour are observed. For *n* = 34 and 35 the measured values of *τ*_decay_ are shorter than those of the surrounding states that follow the general trend. While, for *n* = 40, 43, 45, and 46 the measured values of *τ*_decay_ are longer.

In the hydrogen atom, the fluorescence lifetimes of ℓ-mixed Rydberg states scale approximately with *n*^4^.^77^ Therefore, the observed general reduction in *τ*_decay_ with *n* for long-lived ‘hydrogenic’ Rydberg–Stark states in NO in Fig. 9 indicates that intramolecular interactions with short-lived states must occur. These interactions must in general increase with *n*. However, for particular values of *n* at which deviations from this general trend occur, additional contributions from interactions with more energetically localised states must arise.

### 4.3 Vibrational channel interactions

The general reduction in the measured values of *τ*_decay_ as the value of *n*, and hence *υ*_2_, increase in the data in Fig. 9, occurs because of weak intramolecular interactions with short-lived low-*n* electronic states in series that converge to the X^+ 1^Σ^+^(*v*^+^ = 1, *N*^+^) state of NO^+^. As can be seen in the energy-level diagram in Fig. 2, *n* = 7 states in the *v*^+^ = 1 series lie above, but close to, the *v*^+^ = 0, *N*^+^ = 0 series limit. The lifetimes of these states in the *v*^+^ = 1 series are dominated by fast non-radiative decay processes, *i.e.*, a combination of predissociation and vibrational autoionisation.^44^ Transitions to these states have spectral widths of ∼5 cm^−1^, and hence they are estimated to have lifetimes of ∼1 ps.^44^ Studies have been reported previously of the Δ*v*^+^ ≠ 0 vibrational channel interactions in NO between Rydberg states in series converging to the *v*^+^ = 1 series limit, and the *v*^+^ = 0 continuum that give rise to autoionisation.^44,78^ In the case of interest here these same vibrational channel interactions are seen to persist between states with *v*^+^ = 0 and *v*^+^ = 1 below both series limits.

To evaluate the effects of these vibrational channel interactions in the experiments here, calculations of *τ*_decay_ were performed including contributions from the *v*^+^ = 0 fluorescence and dissociation decay rates, as described in Section 3.3. In these calculations the interactions between states in the *v*^+^ = 0 and *v*^+^ = 1 series were treated semi-empirically. The amount of low-*n*, *v*^+^ = 1 character mixed into a *v*^+^ = 0 Rydberg–Stark state |*i*〉 was described by the value of a Lorentzian function,22
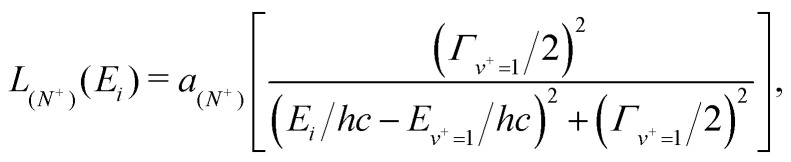
at the off-resonance wavenumber *E*_*i*_/*hc* of the Rydberg–Stark states for which the trap decay time was measured. *E*_*v*^+^=1_/*hc* is the wavenumber of the peak of the Lorentzian function, *Γ*_*v*^+^=1_ is the *v*^+^ = 1 spectral width (*Γ*_*v*^+^=1_ = 1/2π *c τ* _*v*^+^=1_ where *τ*_*v*^+^=1_ ∼ 1 ps), and *a*_(*N*^+^)_ is a scaling factor. In the interpretation of the experimental data in Fig. 9, *E*_*v*^+^=1_ and *a*_(*N*^+^)_ were chosen to be fit parameters. Their values depended on the dominant *N*^+^ character of the state |*i*〉. The total decay rate, *γ*_tot(*i*)_, of the state |*i*〉 was then considered to be,23
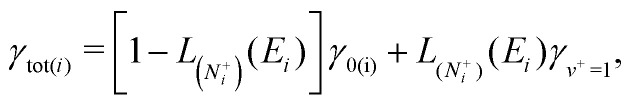
where *γ*_*v*^+^=1_ = 1/*τ*_*v*^+^=1_ is the *v*^+^ = 1 decay rate, and *γ*_0(*i*)_ is given by eqn (17). The decay time constant of the state |*i*〉 was then *τ*_tot(*i*)_ = 1/*γ*_tot(*i*)_. This approach allowed the *v*^+^ = 1 character of the *n*(*N*^+^) Stark states prepared in the experiments to be estimated.

Because the data in Fig. 9 were recorded for times greater than 250 μs after laser photoexcitation, the decay rates after this time were calculated using a similar procedure to that employed by Bixon and Jortner.^43^ This involved considering the population of all accessible short and long lived *n*(*N*^+^) Stark states, each of which decay exponentially. The resulting exponential functions were summed,24
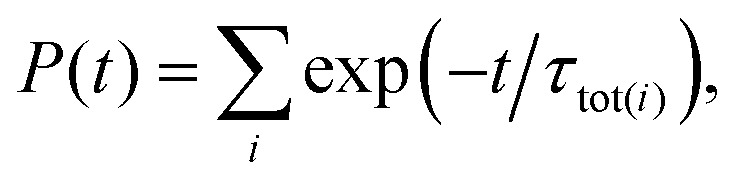
to give the total Rydberg state population, *P*(*t*), where *t* = 0 μs corresponds to the photoexcitation time. A single exponential function was then fit to the results of these calculations between *t* = 300 μs and 600 μs, *i.e.*, between the times corresponding to *t*_trap_ = 50 to 350 μs. Because the deceleration and trapping process only accepted the outer LFS Rydberg–Stark states, this procedure was implemented for the outer quarter of the Stark manifold for each value of *n*(*N*^+^) to obtain the calculated time constant *τ*_calc_.

Calculations of *τ*_calc_ were performed using fixed values of the electric field, *F*_*z*_, and *M*_*N*_. The value of *F*_*z*_ was taken to be equal to the time-averaged field experienced by the molecules in the electrostatic trap obtained from numerical particle trajectory calculations.^47^ For *V*_0_ = 149 V this field was *F*_*z*_ = 95 V cm^−1^, and was approximately constant across the range of values of *n* studied. The values of *M*_*N*_ were restricted to |*M*_*N*_| > |*N*^+^ − 3| such that at least one of the 2*N*^+^ + 1 Stark manifolds, for each *n*(*N*^+^), contained no short-lived ℓ ≤ 3 character, *i.e.*, for *N*^+^ = 0, 1, and 2, |*M*_*N*_| ≥ 4, 3, and 2, respectively.

**Fig. 10:**
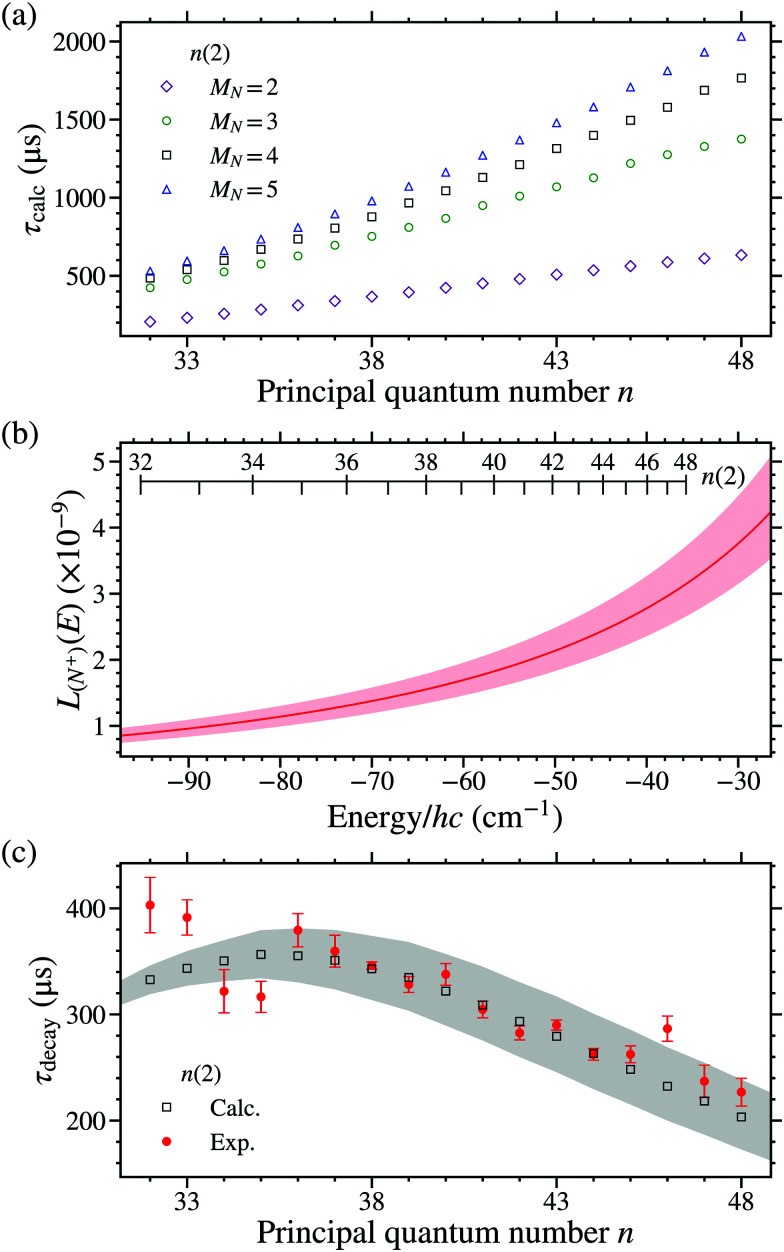
(a) Calculated decay time constants, *τ*_calc_, excluding effects of vibrational channel interactions for *M*_*N*_ = 2, 3, 4, and 5 LFS *n*(2) Rydberg–Stark states in NO. (b) Fraction of *v*^+^ = 1 (*τ*_*v*^+^=1_ ∼ 1 ps) character mixed into the *n*(2) *v*^+^ = 0 Stark states. The red shaded region represents the uncertainty in the fit parameters (see text for details). (c) Comparison of the experimentally measured values of *τ*_decay_ for *n*(2) states (filled red circles) with calculated values of *τ*_calc_ for LFS *n*(2), *M*_*N*_ = 4 Stark states including effects of vibrational channel interactions (open black squares). The grey shaded region represents the uncertainty in *τ*_calc_ resulting from the uncertainty in the fit parameters.

The decay time constants, *τ*_calc_, where first calculated without including effects of vibrational channel interactions (*i.e.*, *a*_(*N*^+^)_ = 0 in eqn (22)). In this case only the intrinsic *v*^+^ = 0 fluorescence and dissociation decay rates were considered. For the *n*(2) *v*^+^ = 0 states, the resulting time constants are shown for *M*_*N*_ = 2 to 5 in Fig. 10(a). From these data it is seen that when only *v*^+^ = 0 fluorescence and dissociation decay rates were included the value of *τ*_calc_ increases with *n*, as would be expected in an atom. This does not however reflect the general trend observed in the experimental data in Fig. 9.

To determine the values of *E*_*v*^+^=1_ and *a*_(*N*^+^)_ for each *N*^+^ series, a fit was performed of *τ*_calc_ to *τ*_decay_. In this fitting process the reduced chi-squared 
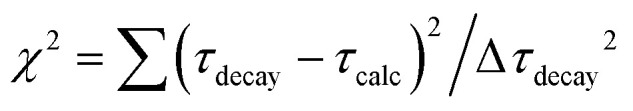
 was minimised in each *N*^+^ series. For *N*^+^ = 0 and 1, all measured values of *n* were used in the fitting process. For *N*^+^ = 2, the fitting was performed for the data points between *n* = 36 and 48 where the values of *τ*_decay_ change smoothly. The values of *τ*_decay_ for the 40(2), 43(2), 45(2), and 46(2) states were not included in the fit because of their deviation from the general trend followed at surrounding values of *n*. A value of *M*_*N*_ = 4 was used in determining *τ*_calc_ because it is the smallest value of *M*_*N*_ for which all *N*^+^ series considered contain at least one set of Stark states without any short lived low-ℓ (ℓ ≤ 3) character. The values of *E*_*v*^+^=1_ and *a*_(*N*^+^)_ which resulted in the minimum chi-squared, *χ*^2^_min_, can be seen in Table 3. The uncertainties on these values were calculated by considering the change required in *E*_*v*^+^=1_ or *a*_(*N*^+^)_ to increase the chi-squared to *χ*^2^ = *χ*^2^_min_ + 1.

**Table 3:** Parameters associated with the Lorentzian function in eqn (22), representing the strength of the vibrational channel interactions between the short-lived (*τ*_*v*^+^=1_ ∼ 1 ps) low-*n* states with *v*^+^ = 1 and the high Rydberg states with *v*^+^ = 0, obtained by minimising the reduced chi-squared of *τ*_calc_ with respect to *τ*_decay_ for the different *N*^+^ hydrogenic series separately. The fitting process was performed with the data recorded for *V*_0_ = 149 V. Values of *M*_*N*_ = 4 and *F*_*z*_ = 95 V cm^−1^ where used in the calculations

*N* ^+^	*E*_*v* ^+^=1_/*hc* (cm^−1^)	*a*_(*N*^+^)_/10^−6^
0	−7 ± 7	0.18 ± 0.08
1	45 ± 10	3.0 ± 0.7
2	31 ± 3	2.0 ± 0.2

Comparison of the best-fit values of *E*_*v*^+^=1_ in Table 3 with the energy-level structure in Fig. 2 shows that the peaks of the Lorentzian functions, which approximately represent the positions of the *v*^+^ = 1 (*τ*_*v*^+^=1_ ∼ 1 ps) states mixed into the *v*^+^ = 0 Stark states, lie close to or above the *v*^+^ = 0, *N*^+^ = 0 series limit and are in the vicinity of the calculated energies of the (*n* = 7)X^+ 1^Σ^+^(*v*^+^ = 1, *N*^+^) states. From the values of *E*_*v*^+^=1_ and *a*_(*N*^+^)_, in Table 3 and eqn (22) it can be estimated that the fraction of short lived (*τ*_*v*^+^=1_ ∼ 1 ps) *n* = 7, *v*^+^ = 1 character mixed into the *n*(*N*^+^) *v*^+^ = 0 Stark states is between 10^−10^ and 10^−9^. For example, a 10^−9^ contribution from a short-lived state with a lifetime of 1 ps, mixed into a long-lived state with a lifetime of 1 ms, yields a total decay rate of 2000 s^−1^ and a lifetime of 500 μs.

Fig. 10(b) shows the fraction of (*n* = 7)X^+ 1^Σ^+^(*v*^+^ = 1, *N*^+^) character mixed into the predominantly *v*^+^ = 0, *N*^+^ = 2 Rydberg states below the *v*^+^ = 0, *N*^+^ = 0 series limit as given by eqn (22). The shaded region corresponds to the range of mixing fractions that arise from the uncertainty in the fit parameters. As can be seen in Fig. 10(b) the fraction of this *n* = 7, *v*^+^ = 1 character mixed into the *n*(2) Stark states increases by approximately a factor of 5 over the wavenumber range studied. In Fig. 10(c) the effects of these vibrational channel interactions on the values of *τ*_calc_ for the *n*(2) states (black squares) can be seen, and these results are compared to the measured values of *τ*_decay_ (red circles). The grey shaded region in this panel of Fig. 10 corresponds to the uncertainty in *τ*_calc_ arising from the uncertainty in the fit parameters. The calculated decay time constant obtained for the 38(2), *M*_*N*_ = 4 [43(2), *M*_*N*_ = 4] state using this approach was *τ*_calc_ = 343 ± 30 μs [*τ*_calc_ = 279 ± 35 μs]. It can be seen in Fig. 10(c) that, in contrast to the case in which only *v*^+^ = 0 fluorescence and dissociation rates were considered [Fig. 10(a)], this type of vibrational channel interaction leads to a dependence of the excited state decay times on the value of *n* that follows the trend observed in the experiments. The contributions from this interaction to the excited state decay rates are ∼1 kHz.

To further validate this interpretation of the experimental observations as arising from vibrational channel interactions, a two state quantum mechanical model was implemented. To approximate the interaction between a *v*^+^ = 0 and *v*^+^ = 1 state, a 2 × 2 Hamiltonian, *H*_vib_, was constructed,25
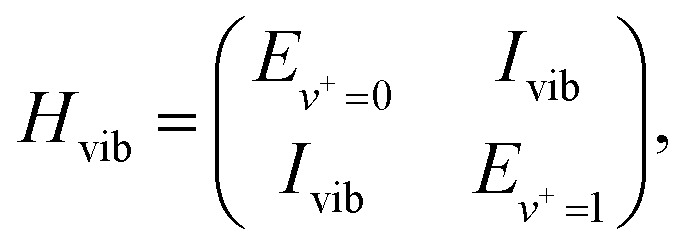
where *E*_*v*^+^=0_ and *E*_*v*^+^=1_ represent the energies of the *v*^+^ = 0 and *v*^+^ = 1 states, respectively, and *I*_vib_ represents the strength of the interaction. Within the harmonic oscillator approximation, Δ*v*^+^ = 1 mixing occurs when an interaction is present that causes the Hund's-case-(b) quantum defects to have a non-zero derivative with respect to the internuclear separation *R*, when evaluated at the equilibrium internuclear distance *R*_e_, *i.e.*,^44,63,79–82^26
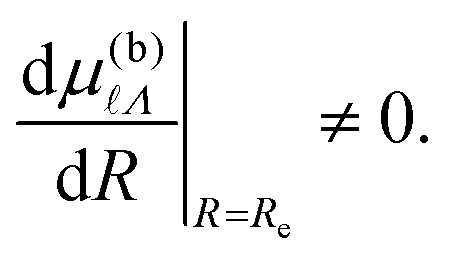
For the Rydberg states of NO the dependence of *μ*^(b)^_ℓ*Λ*_ on *R* arises as a result of the interaction of the Rydberg electron with the *R*-dependent multipole moments of the NO^+^ ion core.^44,59^ The off-diagonal matrix elements associated with this interaction, *I*_vib_ in eqn (25), can therefore be estimated by considering the interaction between the |*n*ℓ*N*^+^*NM*_*N*_*v*^+^ = 0〉 and |*n*′ℓ′*N*^+^′*N*′
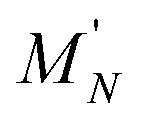
*v*^+^′ = 1〉 Hund's-case-(d) basis states. This interaction can be expressed as,^63^27
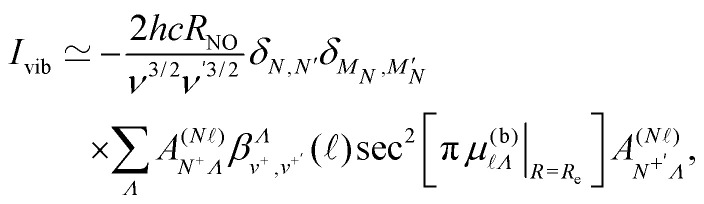
where, for Δ*v*^+^ = 1,28
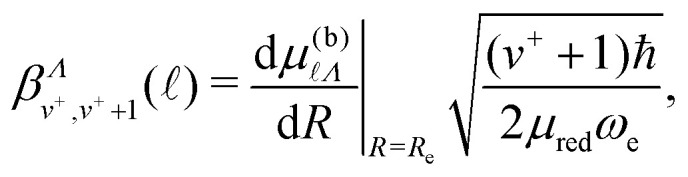
with *μ*_red_ and *ω*_e_ the reduced mass and vibrational frequency of NO^+^, respectively. The units of the coupling given by eqn (27) are introduced through the density of Rydberg states as in the first order approximation to MQDT.^52,80^ It is assumed that the values of the Hund's-case-(b) quantum defect at the equilibrium internuclear distance, *μ*^(b)^_ℓ*Λ*_|_*R*=*R*e_, are small because ℓ ≥ 4, and hence 
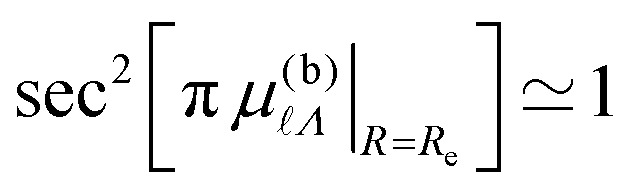
. The derivatives of the quantum defects, for *Λ* ≤ 2, were obtained by considering the low-*n*, *R*-dependent quantum defects calculated in ref. 59. For *Λ* ≥ 3, it was assumed that 
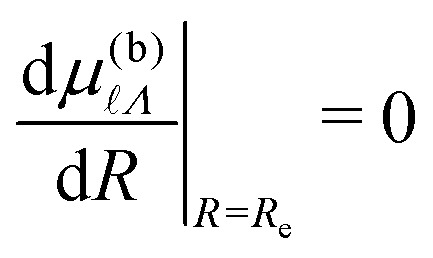
. Values of *I*_vib_ were then determined by considering the interactions between states for which *v*^+^ = 0, *N*^+^ = 2, and *n* = 38, and those for which *v*^+^ = 1, *N*^+^ = 0 and 4, *n* = 7, and ℓ = 4 and 5. Following this approach the interaction strength *I*_vib_ was calculated to be between *I*_vib_/*hc* ≃ 1 × 10^−3^ cm^−1^ and 5 × 10^−2^ cm^−1^. The effect of this interaction on the *v*^+^ = 0 and *v*^+^ = 1 states was then determined by considering that (*E*_*v*^+^=1_ − *E*_*v*^+^=0_)/*hc* ∼ 100 cm^−1^, and calculating the eigenvalues and eigenvectors of the Hamiltonian in eqn (25). The resulting mixed states contain 10^−10^ to 10^−7^ of the minority component, *i.e.*, the absolute values of the amplitudes of the minority component in the wave functions are 10^−5^ to 10^−3.5^. These mixing fractions are consistent with those expected from the measured trap decay times. Using this two-state model it can also be estimated that the vibrational channel interaction considered here would give rise to wavenumber shifts of the *v*^+^ = 0 and *v*^+^ = 1 states of ∼10^−8^ cm^−1^ to 10^−5^ cm^−1^ (*i.e.*, frequency shifts of ∼100 Hz to 100 kHz).

### 4.4 Rotational channel interactions

To interpret the trap decay time constants for the individual Rydberg states that deviate from the general trend in Fig. 9, *i.e.*, that *τ*_decay_ decreases with increasing *n*, it is necessary to consider the role of rotational channel interactions between states with *v*^+^ = 0. As discussed by Bixon and Jortner,^43^ and in Section 4.1, the hydrogenic Stark states populated by photoexcitation in the experiments here are those that undergo intramolecular interactions with nearby optically accessible *n*p(0) or *n*f(2) states. These interactions can be induced solely by external electric-fields, as is the case for *n*(2) states that mix with *n*f(2) states, or by a combination of intramolecular charge-multipole interactions and external electric-fields, that occur, for example, in the case of *n*(1) states populated because of accidental degeneracies between *n*p(0) and *n*′d(1) states (where *n*′ ≠ *n*), with the *n*′d(1) states further mixing with the *n*′(1) hydrogenic states.

In Fig. 9(c) the measured values of *τ*_decay_ for the 40(2), 43(2), and 45(2) Rydberg–Stark states are larger than expected from the behaviour of the surrounding states. These *n*(2) hydrogenic Stark states are populated because of the effects of mixing with the *n*f(2) states. However, the charge-quadrupole interactions that couple states for which Δ*N*^+^ = ±2 and Δℓ = 0 lead to mixing between *n*f(2) and *n*′f(0) states, which, in the presence of an electric field also mix with the *n*′(0) states. This type of interaction with intramolecular and external field contributions leads to the population of *n*′(0) states upon photoexcitation if they are energetically close to an *n*f(2) resonance. Hence, because of accidental degeneracies with *n*′(0) states, photoexcitation on the 40f(2), 43f(2), and 45f(2) resonances leads to the population of both the 40(2) and 44(0), the 43(2) and 48(0), and the 45(2) and 51(0) states, respectively. Since the *n*′(0) states have longer decay time constants than the *n*(2) states at a particular excitation wave number (see Fig. 9), trapped molecules in these states have larger values of *τ*_decay_ than those excited on resonances where only *n*(2) states are populated. This increase in the observed decay time constant also depends on the ratio of the *n*(2) and *n*′(0) state populations.

Further deviations from the general trend in the values of *τ*_decay_ in Fig. 9 are also seen for the 34(2) and 35(2) states. In these cases, charge–dipole interactions that couple states for which Δ*N*^+^ = ±1 and Δℓ = ±1 lead to mixing of the *n*f(2) and *n*′ℓ′(3) states. These result in the population of *n*′(3) states, in addition to *n*(2) states, upon photoexcitation. Consequently, excitation on the 34f(2) and 35f(2) resonances leads to the population of both the 34(2) and 32(3), and the 35(2) and 33(3) states, respectively. The reduced decay time constants observed for *n* = 34 and 35 in Fig. 9(c), when compared to the surrounding states, suggests that the 32(3) and 33(3) states have shorter lifetimes than the 34(2) and 35(2) states.

The measured decay time constant for the 46(2) state in Fig. 9, is larger than expected from the trend in the surrounding states. In this case the presence of both the optically accessible 46f(2) and 53p(0) states within the laser spectral width, and the increasing overlap of the Stark manifolds at these high values of *n* complicate the interpretation of this feature. However, based upon similar arguments to those outlined above, an estimate of the states that can be populated in both of these cases can still be made. Although a rigorous treatment, *e.g.*, by combining particle trajectory calculations with MQDT, will be required to determine the relative populations of each Stark state and the efficiency with which each can be decelerated and trapped. In this instance it is considered that:

• The 46(2) Stark states are populated because of electric-field-induced mixing with the 46f(2) states.

• The 50(1) Stark states are almost degenerate with the 46(2) states and can therefore be populated as a result of charge-dipole interactions that (i) couple the 46f(2) states and 50d(1) or 50g(1) states, or (ii) couple the 53p(0) state with the 50s(1) or 50d(1) states. These are then mixed with the hydrogenic 50(1) states in the electric field.

• Finally, 52(0) Stark states can be populated as a result of electric-field-induced mixing with the 53p(0) state.

Since, at a particular excitation wavenumber the *N*^+^ = 0 Stark states were observed to exhibit longer decay time constants than the *N*^+^ = 1 and 2 Stark states (see Fig. 9), the presence of 52(0) states is expected to increase the value of *τ*_decay_ measured at the wave number associated with the excitation of the 46(2) states when compared to the other surrounding states. This is consistent with the experimental observations.

**Fig. 11:**
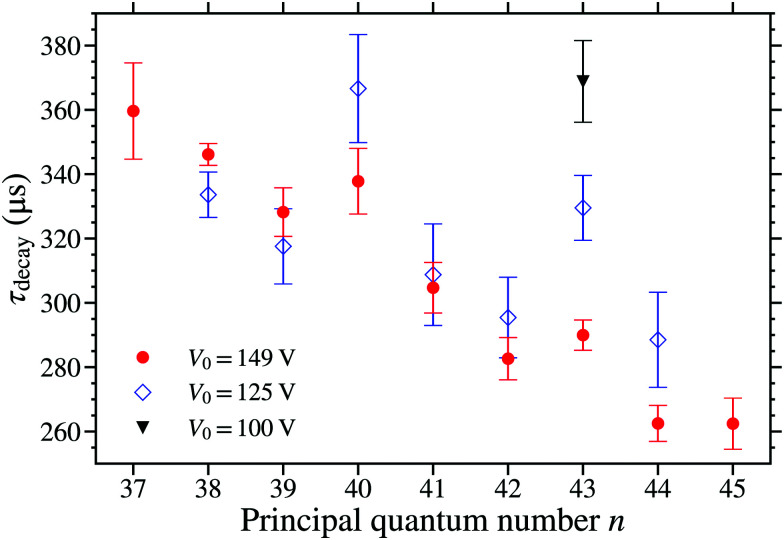
Measured values of *τ*_decay_, for states in the *N*^+^ = 2 series. The data were recorded for *V*_0_ = 100 V (black triangle), 125 V (blue open diamonds), and 149 V (red circles).

The dependence of *τ*_decay_ on the trap depth was measured for the *n*(2) states in the range from *n* = 38 to 44, see Fig. 11. The data in this figure were recorded for three different trap depths with *V*_0_ = 149 V (red circles), *V*_0_ = 125 V (blue open diamonds), and *V*_0_ = 100 V (black triangle). From these data, it is seen that in general *τ*_decay_ is insensitive to the value of *V*_0_, and hence the trap depth. This is consistent with the results of calculations for *F*_*z*_ = 95 V cm^−1^ (*V*_0_ = 149 V) and *F*_*z*_ = 80 V cm^−1^ (*V*_0_ = 125 V) in which the reduction in the electric field strength caused *τ*_calc_ to change by <5 μs over this range of values of *n*. However, two exceptions to this general trend are seen in Fig. 11. These occur for the 40(2) and 43(2) states where the value of *τ*_decay_ increased as the value of *V*_0_ was decreased.

As discussed above, photoexcitation on the 40f(2) and 43f(2) resonances results in the population of the 40(2) and 44(0), and the 43(2) and 48(0) Stark states, respectively. The increase in *τ*_decay_ as *V*_0_ was decreased at these resonances indicates that in these cases the fractions of trapped molecules in the longer lived 44(0), or 48(0), states also increase as *V*_0_ decreases. For states with *n* > 40 the trapping efficiency is limited by electric field ionisation during deceleration and trapping, with the ionising field scaling with *n*^−4^. For lower values of *V*_0_, the electric field experienced by the molecules during deceleration and trapping is reduced. This leads to an increase in the trapping efficiency for molecules in higher *n* states, because of reduced loses from electric field ionisation and the resulting increase in the effective volume of the trap. This increase in trap loading efficiency is greater for states with higher values of *n*. Therefore, for the measurements performed following excitation on the 40f(2) resonance, the trapping efficiency for molecules in the 40(2) states only changes a little between *V*_0_ = 149 V and 125 V, whereas the efficiency of trapping molecules in the near degenerate 44(0) states increases as the values of *V*_0_ is reduced. This results in an increase in the fraction of molecules in the longer-lived 44(0) states present in the trap when *V*_0_ = 125 V, compared with *V*_0_ = 149 V, and therefore *τ*_decay_ is observed to increase. The situation is similar for the 43f(2) resonance, where the trapped molecules are in the 43(2) and 48(0) states, and the fraction of molecules in the longer-lived 48(0) states increases as *V*_0_ decreases resulting in an increase in *τ*_decay_.

## 5 Conclusions

In conclusion, NO molecules have been prepared in high Rydberg states with lifetimes in excess of 100 μs, decelerated to rest in the laboratory-fixed frame of reference in the travelling traps of a transmission-line Rydberg–Stark decelerator and electrostatically trapped. Trapping was performed in a cryogenic environment to minimise the effects of blackbody radiation induced transitions. Measurements, of the decay of the molecules from the trap, were conducted *in situ* by pulsed electric field ionisation. The long lifetimes of the Rydberg states observed in the experiments are reminiscent of those of Rydberg states in atoms. However, over the timescales of ∼1 ms on which the experiments were performed, molecular effects were seen to play a significant role in the decay of the trapped molecules, and result in a breakdown of *n*-scaling rules typical of hydrogenic systems. These effects arise because of rotational and vibrational channel interactions which originate from the coupling of the Rydberg electron to the electric dipole, quadrupole, and higher order multipole moments of the NO^+^ ion core. These types of channel interactions have been discussed previously in the literature, in the context of the lifetimes of Rydberg states in ZEKE spectroscopy experiments. However, ZEKE experiments are generally performed on the 1 μs timescale and many of the effects of these interactions are weak. Consequently, the quantum-state-dependence of each of the interaction mechanisms identified here, which typically contribute ∼1 kHz to the excited state decay rates, were not observable. In the experiments reported here, with measurement times of up to 1 ms, these effects are clearly seen. Vibrational channel interactions between Rydberg states in the *n*X^+ 1^Σ^+^(*v*^+^ = 0, *N*^+^) series, and short-lived (*n* = 7)X^+ 1^Σ^+^(*v*^+^ = 1, *N*^+^) states, lead to a general reduction in the lifetimes of the *n*X^+ 1^Σ^+^(*v*^+^ = 0, *N*^+^) Rydberg states with *N*^+^ = 0, 1, and 2 and values of *n* > 35 as each series limit is approached. Rotational channel interactions within the *v*^+^ = 0 series have been identified to specifically affect the decay time constants of some individual Rydberg states.

The effects of molecular interactions on the lifetimes of Rydberg states studied here must be carefully considered when employing general *n*-scaling rules in the analysis of excited state decay, *e.g.*, following electron–ion recombination. The experimental methods developed and implemented in this work offer opportunities for similar studies with other molecules, *e.g.*, N_2_ and O_2_, provided schemes for populating long-lived Rydberg states are identified. The results reported here will provide important input for future low-energy collision experiments using Rydberg NO molecules, and the implementation of schemes to exploit the technique of Rydberg–Stark deceleration to prepare cold samples of ground state molecules.

## Conflicts of interest

There are no conflicts to declare.

## Supplementary Material
